# A novel CMAQ-CNN hybrid model to forecast hourly surface-ozone concentrations 14 days in advance

**DOI:** 10.1038/s41598-021-90446-6

**Published:** 2021-05-25

**Authors:** Alqamah Sayeed, Yunsoo Choi, Ebrahim Eslami, Jia Jung, Yannic Lops, Ahmed Khan Salman, Jae-Bum Lee, Hyun-Ju Park, Min-Hyeok Choi

**Affiliations:** 1 Departmcnt of Earth and Atmospheric Sciences, University of Houston, Houston, TX 77004 USA; 2 Houston Advanced Research Center, The Woodlands, TX 77381 USA; 3 National Institute of Environmental Research, Incheon, Korea

**Keywords:** Environmental sciences, Mathematics and computing

## Abstract

Issues regarding air quality and related health concerns have prompted this study, which develops an accurate and computationally fast, efficient hybrid modeling system that combines numerical modeling and machine learning for forecasting concentrations of surface ozone. Currently available numerical modeling systems for air quality predictions (e.g., CMAQ) can forecast 24 to 48 h in advance. In this study, we develop a modeling system based on a convolutional neural network (CNN) model that is not only fast but covers a temporal period of two weeks with a resolution as small as a single hour for 255 stations. The CNN model uses meteorology from the Weather Research and Forecasting model (processed by the Meteorology-Chemistry Interface Processor), forecasted air quality from the Community Multi-scale Air Quality Model (CMAQ), and previous 24-h concentrations of various measurable air quality parameters as inputs and predicts the following 14-day hourly surface ozone concentrations. The model achieves an average accuracy of 0.91 in terms of the index of agreement for the first day and 0.78 for the fourteenth day, while the average index of agreement for one day ahead prediction from the CMAQ is 0.77. Through this study, we intend to amalgamate the best features of numerical modeling (i.e., fine spatial resolution) and a deep neural network (i.e., computation speed and accuracy) to achieve more accurate spatio-temporal predictions of hourly ozone concentrations. Although the primary purpose of this study is the prediction of hourly ozone concentrations, the system can be extended to various other pollutants.

## Introduction

Surface ozone can pose a significant health risk to both humans and animals alike, and it also affects crop yields (USEPA—2006). According to the US Clean Air Act, it is one of the six most common air pollutants and considering its impact on health, the Environmental Protection Agency (EPA) of the United States has limited the maximum daily eight-hour average (MDA8) concentration of ozone to 70 ppb. Similarly, the Ministry of Environment in South Korea has declared a standard for hourly ozone of 100 ppb and 60 ppb for MDA8. To achieve these attainment goals and to understand future projections (forecasts), researchers have turned to various numerical modeling and statistical analysis tools. One such numerical model is the Community Multi-scale Air Quality Model (CMAQ), a chemical transport model (CTM) developed by the USEPA^1^. Widely used to forecast the air quality of a region with considerable accuracy, CMAQ is an open-source multi-dimensional model that provides estimated concentrations of air pollutants (e.g., ozone, particulates, NO_x_) at fine temporal and spatial resolutions. It has been used as a primary dynamical model in regional air pollution studies; CMAQ modeling, however, has several limitations (e.g., parameterization of physics and chemistry) and raises uncertainties that lead to significant biases (overestimations or underestimations) overestimations of ozone concentrations^2–6^. Also, the stochastic nature of the atmosphere results in inherent uncertainty in even comprehensive models that might limit their accuracy^7^.

CTMs require substantial computational time since they entail multiple physical and chemical processes for each grid. The testing time for various scenarios of the CMAQv5.2beta configuration test (with US EPA Calnex 12 km domain July 2, 2011 testing dataset) was in the range of 34–54 min (Further detail about the test can be found at CMAQ version 5.2beta (February 2017 release) Technical Documentation—CMASWIKI (airqualitymodeling.org))^8^. Unlike CTMs, machine learning (ML) can be trained to forecast multi-hour output using a certain set of inputs more accurately within faster processing time^9,10^. In addition, it requires only one training process, further reducing the computational time. Although all ML models are more accurate with faster processing speeds, they are very localized (station-specific) and generate large underpredictions of daily maximum ozone concentrations^9,11,12^.

The objective of using this ML technique is to enhance the CMAQ modeling results by taking advantage of (1) the deep neural network (DNN), a computationally efficient, artificially intelligent system that recognizes uncertainties resulting from simplified physics and chemistry (e.g., parameterizations) of the CMAQ model; and (2) CMAQ, which computes unmeasured chemical variables along with fine temporal and spatial resolutions. The aim of this approach is to use the best of both numerical modeling and ML to design a robust and stable algorithm that more accurately forecasts hourly ozone concentrations 14 days in advance and covers a larger spatial domain. The ML technique used in this study was based on the convolutional neural network (CNN) model.

## Discussion

We trained the models based on two loss functions (methods 1 and 2) and fourteen days (28 different models), from January 1, 2014, 0000UTC to December 31, 2016, 2300UTC. After training the models, we evaluated them based on various performance parameters. The index of agreement (IOA) based on hourly values of the year 2017 was calculated for each station and then averaged. (The IOA was selected over correlation as the performance metric for reporting the results because (1) a correlation of 1 doesn’t mean that model captures the high and lows; (2) an IOA considers the bias within the performance metric. Thus, an IOA of 1 will mean that all highs and lows of a time series were captured well. Furthermore, the numerator of IOA addresses the mean-bias (supplementary document: General Statistical Analysis)). The models based on both methods of the CNN model reported the highest IOA for predicting one day ahead, but the IOA decreased on subsequent days. The average IOAs (method 1—0.90, method 2—0.91) and correlations (method 1—0.82, method 2—0.83) for one-day ahead prediction were comparable. The performance of both methods showed improvement over that of the CMAQ model (IOA-0.77, correlation-0.63). The IOA with method 1 increased by 16.86% and that with method 2 by 17.98%. The correlation with method 1 increased by 30% and that with method 2 by 32%.

### Performance comparisons of CMAQ and CNN models

**Figure 1:**
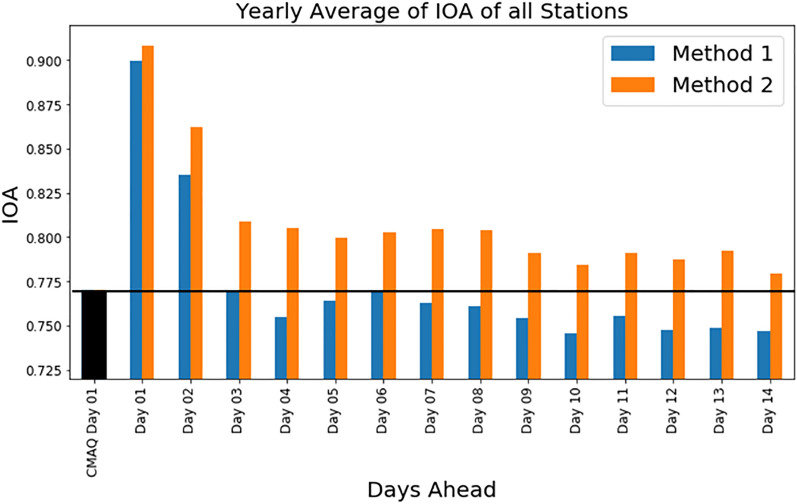
Comparison of Index of Agreement for two-advance prediction using Method 1 and 2. x-axis in the plot shows the days ahead, and the y-axis represents the index of agreement. The blue line represents the IOA of each day's advance prediction using Method 1 (mean squared error as loss function). The orange line represents the IOA of each day advance prediction using Method 2 (Index of Agreement as loss function).

Figure 1 shows the yearly IOA (average of all stations). The IOA decreased sharply from day 1 to day 3 but stabilized after the three-day forecasts from both methods. The IOA for day 4 was lowest during the first week of prediction for method 1. After day 4, the IOA increased until day 6 and then decreased until day 10. It increased slightly on day 11 but then decreased further. For method 2, the IOA decreased until day 5, increased until day 7, and then further decreased after day 8. One possible explanation for the weekly trend relates to the weekly cycle of ozone concentrations^13^. Figure S1 (Supplementary Document) shows the autocorrelation (average of all stations) of the current day observed ozone with the subsequent day observations [The autocorrelation is the correlation of current hourly values with subsequent hours (0 to 336 h)]. The observed ozone followed a weekly cycle, exhibiting a decreasing trend in its correlation until day 3 and then an increasing trend until it peaked on day 7. The same cycle occurred during the second week. From Fig. 1 and figure S1, it is evident that the CNN model also follows this weekly trend. Also, Fig. 1 depicts the superior performance of the CNN model method 2 to that of method 1. The average increase in the IOA of method 2 compared to that of method 1 was 4.77%; a maximum increase of 6.64% occurred on day 4, and a minimum increase of less than 1% occurred on day 1. The greatest increase in the IOA happened on the worst-performing days (days 4, 13,8, 7, and 12 show an increase of 6.6, 5.8, 5.6, 5.4, and 5.3%, respectively) by method 1.

Figure S2 to S7 (Supplementary Document) shows the hourly time series plots for the month of February and June of stations 131,591, 238,133, and 823,691 (The stations shown here have the highest, median, and least IOA for Day 1 forecasts by the CNN-method 2 model). These figures show that both the CNN models are good up to 7 days forecast. For seventh- and fourteenth-day forecasts, the CNN-method 2 model performed better than the method 1 model. Even though the performance decreases, the CNN models can produce a reliable forecast for up to 14 days.

In terms of mean bias (Table S1—Supplementary document), both the CNN models were under predicting for all days while the CMAQ model for the day 1 forecast was overpredicting. The average of all stations' mean bias for the CMAQ forecast was 1.21. Whereas for CNN-method 1 and CNN-method 2 models, the mean bias was − 1.23 and − 0.96, respectively. From day 2 onwards, the model, with IOA as loss function, performed significantly better than the model with MSE as loss function. The root means square error (RMSE) for the CMAQ model, the CNN model method 1, and the CNN model method 2 were 18.98, 11.00, and 11.01 for day 1 forecasts, respectively (Table S2—Supplementary document). While for day 1, both the methods of the CNN model were equivalent for RMSE, in subsequent days, CNN-method 2 performed slightly better than CNN-method 1. In terms of correlation (Table S3—Supplementary document), method 2 of the CNN model outperformed method 1 as well as the CMAQ model. The correlation for all 14 days forecast by the CNN-method 2 model was greater than or equal to the CMAQ’s forecast for the first day.

The models were also evaluated based on categorical statistics^14,15^ as defined in Chai et al. (2013). For categorical statistics, the National Ambient Air Quality Standards (NAAQS) for daily maximum 8-h average (MDA-8) was used. The standard threshold of 70 ppb was too high to properly evaluate model performance with categorical statistics. Thus, the standard (threshold) was further reduced to 55 ppbv as done in Sayeed et al. (2020)^9^. Tables S4 to S8 in the supplementary document show the hit rate (HIT), false alarm rate (FAR), critical success index (CSI), equitable threat score (ETS), and proportion of correct (POC), respectively. The hit rate for the CNN-method 2 increased to 0.80 from 0.77 for the CMAQ model, while it decreased to 0.67 for the CNN-method 1. The hit rate for day 2 to day 14 forecast ranges between 0.47 and 0.74. The FAR for the CNN-method 1 was better than the CNN-method 2 for all forecast days. The decrease in FAR for the method 1 and 2 were ~ 46% and ~ 35% respectively when compared with the CMAQ model for the first-day forecast. POC, ETS, and CSI were used to evaluate model skills. While the CNN- method 2 performed equivalent to method 1 for POC for all 14 days of forecasts, it had better skill in terms of ETS and CSI when compared with method 1.

From the discussion above, it can be concluded that by changing the cost/loss function, better optimization can be achieved. Introducing IOA as the cost function (see equation 1 in Supplementary Document) not only reduced the bias but also increased the correlation and IOA. This improvement is attributed by the reduction of bias in both high and low values rather than the average/overall bias. Compared to correlation, IOA is a better metric to evaluate a time series since correlation only considers the shape of the time series and not the bias.

### Performance evaluation of selected method

**Figure 2:**
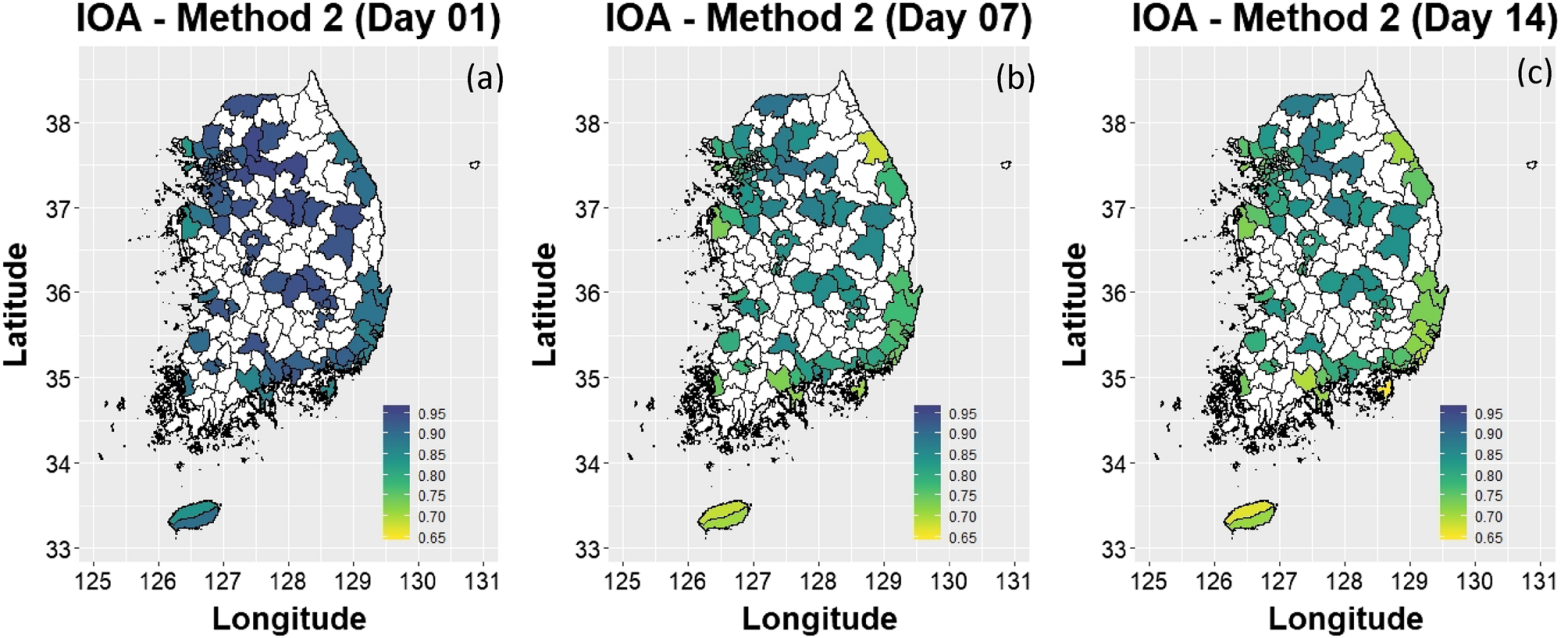
Average IOA all stations (CNN-method 2) in each district of South Korea. (**a**) IOA for Day 1; (**b**) IOA for Day 7; and (**c**) IOA for Day 14. (Figures are created using R ggplot2^27^: https://ggplot2.tidyverse.org/).

**Figure 3:**
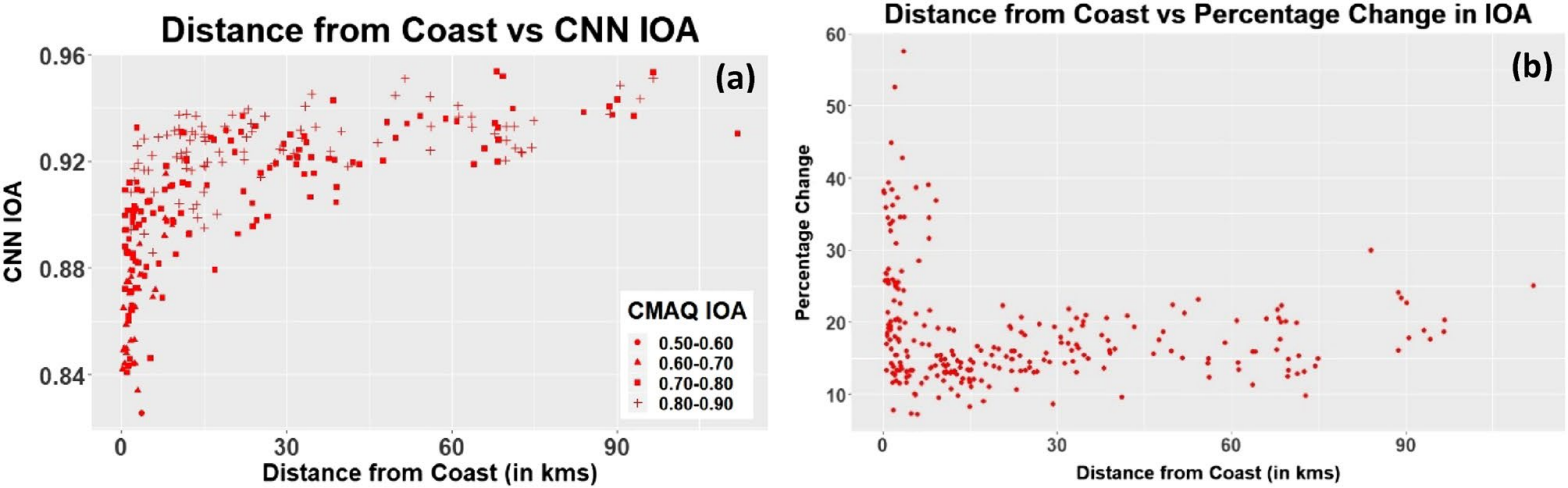
(**a**)Variation of IOA based on distance from the coast. The x-axis represents the distance of the station from the coast, and the y-axis represents the index of agreement. The colored symbols represent the range of CMAQ-IOA for the corresponding station. All IOA are based on one-day ahead prediction only. (**b**) Percentage change in IOA based on distance from the coast. The figure shows the percentage increase in IOA of the CNN model-method 2 when compared with the IOA of the CMAQ model.

It is evident from the above discussion that the performance of the CNN-method 2 overshadowed that of the CNN-method 1; therefore, we further analyze the performance of method 2 below. Figure 2 lists the average yearly IOA of each district in South Korea (Figures were created using R ggplot2 package: https://ggplot2.tidyverse.org/). If a district had more than one station, we averaged its IOAs. We found that inland cities performed slightly better than the coastal ones, and their performance improved the farther they were from the coast (Figs. 2, 3a and Figure S8 in the supplementary document). For example, Seoul performed slightly better than Incheon, the former being farther away from the coast. One explanation for the better performance in the central region is that it has ozone chemistry exhibiting a typical diurnal ozone cycle throughout the year than the coastal region, where predominant land-sea breezes may have an impact on ozone chemistry (Figure S9 in the supplementary document shows 24-h observed ozone concentrations throughout the year. Figures S9-a, b, and c display the three worst-performing stations, while Figures S9-d, e, and f display the three best)^16,17^. It is evident from the figures that stations with the typical diurnal ozone cycle^18^ provided more accurate forecasts than those with less variability in hourly concentrations. Ideally, the ozone concentration starts to increase in the afternoon and peaks a few hours before sunset^11^. This forms a distinct diurnal cycle of ozone concentration (addressed as typical diurnal ozone cycle in this study). The CNN model also follows this typical ozone chemistry and attempts to make predictions based on this information; hence, the station with generalized (typical) ozone chemistry produced more accurate forecasts than the station with less variability in its concentration of ozone throughout the day (Since the sample size of the typical ozone diurnal cycle was much greater than the diurnal cycle with less variability, the CNN model was biased toward the former.)

The accuracy of forecasting was also dependent on the level of urbanization (Figs. 2 and S10 in the supplementary document). Out of seven cities with an IOA higher than 0.94, six were among the least urbanized (the 4th and 5th quantiles: urbanization quantiles based on Chan et al. (2015)^19^), and only one was an urban region (the 2nd quantile). Ozone precursors are mostly anthropogenic in urban areas that can be highly variable^13^. This variability leads to a departure from the general (or ideal) diurnal trend of ozone concentrations and thus leads to less accurate forecasting of method 2 in urban areas than in rural areas.

Figure 3a shows station-wise IOA based on the distance from the coast. The symbol in the figure represents the bin of the CMAQ model’s IOA. It is evident from the figure that the IOA for the CNN model- method 2 increases as we move further away from the coast. The possible reason for the low IOA is that the CMAQ itself has a lower IOA near the coast. From Fig. 3b, it is evident the stations closest to the coast show more improvement compared to the stations away from the coast, in general. The increase in IOA with the CNN model—method 2 was in the range of 7–55% when compared with the CMAQ model for day 1 forecast (Figure S11 – Supplementary Document shows the station-wise IOA for the CMAQ and the CNN-method 2 model 1 day forecast). Among the stations on the coastal regions, those on the northwestern coast provided less accurate predictions than those on the northeastern and southeastern coastal cities (Figure S8). A possible explanation for such a trend could be the variability induced by long-range transport from China^20^. The effects of transport are observable at the three stations on Jeju Island. Because of transport from the Korean Peninsula, two stations (339,111 and 339,112) on the northern coast have a lower IOA (0.84 for both stations) than the one station (339,121) on the southern coast (IOA—0.90). As a mountain range separates the northern part of the island from the south, transport is blocked. Note: Location of stations can be found in Figure S12—Supplementary Document.

**Figure 4:**
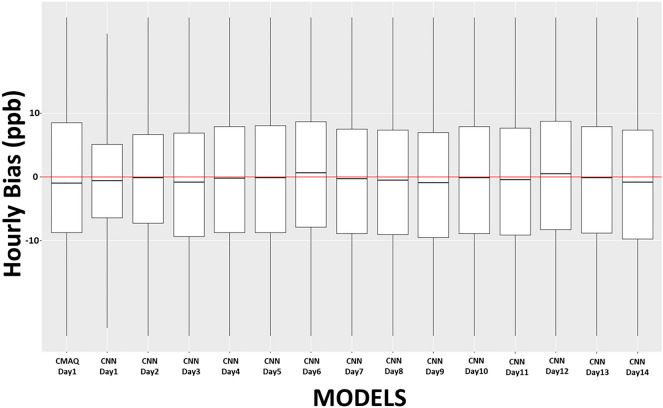
Box plot of hourly bias of all stations combined. The x-axis represents the prediction days, and the y-axis represents the hourly bias in ppb. The Redline represents the zero bias, and the black horizontal line in each box represents the mean bias for that model.

Figure 4 shows the boxplot for the hourly bias of all the stations combined for 14-day advance prediction. The bias for one-day advance prediction using the CNN model -method 2 is the least. As the number of advance prediction days increases, variability in the bias also increases, but the mean bias remains close to 0 for all days. The day 14 forecast has a similar bias as the one-day advance forecast by the CMAQ model. Although the mean bias remains close to zero, it should not be inferred that model performed similarly for all days. The bias resulting from a low observed value will be low, and the frequency of occurrence of a low value in a typical diurnal hourly ozone cycle is more frequent than the high value (Highs occurs only for 4–6 h in a 24-h period). Therefore, the evaluation of an air-quality model must be performed in conjunction with other metrics like IOA, correlation, or both. This is demonstrated in Figure S13 (Supplementary Document), which shows box plot of bias for daily maximum of ozone concentration for all stations (CNN method 2). The interquartile range (IQR) of bias for CNN Day-1 was lower compared to the CMAQ Day-1 bias. The IQR increases with subsequent days and for the 14th day the mean of bias was − 4.89 ppb. From the second day on, the CNN model initiated over predictions, which peaked around days 3and 4 and then began to decrease. Days 7 and 8 showed the fewest over predictions, and the mean of maximum daily ozone was close to the mean of the observations. The second week followed the same trend as that of the first week. Overprediction increased until the 9th and 10th days, and it decreases. The reason for such weekly trends in the IOA of prediction is that ozone concentrations also followed a weekly trend^13^. Ozone concentrations were strongly auto-correlated with the seventh day, which provided better training of the CNN model for days 7 and 14; hence, the performance of the model on these days improved.

## Conclusion

The predictive accuracy of the CNN model depended on one or a combination of multiple factors: (1) the performance of the base model (in this case, CMAQ), (2) distance from the coast, (3) level of urbanization, and (4) transport. These factors, individually or in combination, led to a departure from typical diurnal ozone trends. As a result, an anomaly occurred, and in some cases, the model was not able to successfully understand the anomaly, which led to comparatively less forecasting accuracy. The model generally performs better when the CMAQ performs well. The quantification of variance in performance can provide a future direction for improving the performance further.

The variability caused by the cyclic reversal of land and sea breeze in ozone concentrations led to poor performance by the CNN model in the coastal region. Distance from that coast has an inverse effect on the prediction accuracy of this CNN model. As we move inland, its accuracy improves. Similarly, as a less urbanized locale has a more consistent diurnal ozone trend, training of the CNN model becomes easier, enhancing its prediction accuracy.

The highly contrasting performance of the model, when applied to the western and eastern coasts of South Korea, suggests that transport also plays a significant role in determining the accuracy of model predictions. Unlike the east coast, the western coast is subject to long-range transport that adds to the variability of ozone trends. This hypothesis was supported by observations of the effects of transport at the three stations on Jeju Island.

Apart from affecting individually, the combination of these factors can also lead to poor performance. The low correlation over the northwest coast regions near Incheon is possibly from the combination of land-sea breeze and poor emission inventory. The airport located in Incheon is well-known for missing the NOx source (aircraft emission) in the emission inventory and thus affecting the CMAQ’s performance. Seosan is a big industrial region located south of Incheon where both land-sea breeze and the NOx chemistry affect the typical ozone diurnal cycle. Also, the KORUS-AQ report mentioned that the emission data highly underestimated VOC emission over the region) which is another reason for the poor performance by the CMAQ model and the CNN model.

The developed CNN model was successful in reducing the uncertainties arising from the systematic biases in the system while, to some extent, unable to account for the uncertainties arising from high variability in the atmospheric dynamics, emission, and chemistry. The current systems for air quality prediction are either a short-term forecasting system or a low-accuracy system that covers a more extended forecasting period. Since this model provides a reasonable forecast two weeks in advance, it can provide an actionable window within which government agencies can deploy effective measures for reducing the occurrence of extreme ozone episodes.

## Methods

The proposed algorithm uses two sets of inputs: (1) parameters predicted by numerical models and (2) the previous day's observed air quality.

### Coupled CMAQ and WRF

To take advantage of numerical modeling, we used air quality and meteorological parameters prepared by the CMAQ v5.2^1^ and the Weather Research and Forecasting (WRF) v3.8, covering the eastern part of China, the Korean Peninsula, and Japan, with a 27 km spatial resolution. The detailed configurations of the CMAQ and WRF models are available in Jung et al. (2019)^21^.

### Deep convolutional neural network

We use the deep architecture of the convolution neural network (CNN) used in Sayeed et al. (2019)^9^. The model consists of five convolutional layers and one fully connected layer. We apply convolution to the input features and the elements of the kernel. The final feature map obtained at the end of the first layer of the CNN acts as input for the second layer. Similarly, the output feature map of the second layer is input for the third layer, and so on. In this way, the model has a five-layer CNN, each layer with 32 filters (activation by ReLU), each with a size two kernel randomly initialized by some value for the first iteration. After determining the last feature maps in the last convolutional layer, the fully connected hidden layer with 264 nodes provides the 24-h output of ozone concentrations. We implemented the algorithm in the Keras environment with a TensorFlow backend^22,23^ (Figure S14 in the supplementary document displays a schematic of the deep CNN architecture for the prediction of hourly ozone concentrations for the next fourteen days.)

A deep CNN, like any neural network, is an optimization problem that attempts to minimize the loss function. The most generally used loss functions are the mean squared error, the mean absolute error, and the mean bias error. In this study, we tested two loss functions: (1) the mean square error (method 1) and (2) a customized loss function (method 2) based on the index of agreement (IOA)^24,25^. Mathematical expression of IOA appears in the supplementary section. In method 1, the model attempts to find a solution iteratively such that the mean square error is a minimum. Similarly, in method 2, the model attempts to fit it in such a way that the IOA is maximum. In both cases, we obtain two separate models for each day of prediction. The reason for choosing the IOA as a loss function is that high peaked concentrations in air quality forecasting prediction are critical, and IOA, unlike the mean bias or the mean square error, is a better parameter that more accurately reports the quality of a model. The CNN, like any ML technique, is an optimization problem; the model tries to simulate as close to true observations as possible and relies on minimization or maximization of certain performance parameters. In general, ML algorithms, “mean squared errors” (method 1) are used as the cost (loss) function and the model tries to minimize this loss. The issue with this method in the hourly forecast is that it generalized the model and was unable to predict the high peak values because of the sampling bias (only 3–4 high values in 24-h as compared to 20–21 low or average values). In order to mitigate this issue, we used Index of agreement (IOA) as the cost (loss) function and found out that the model was able to predict better high peaks as compared to method 1.

### Data preparation and model training

We obtained observed air quality from the Air Quality Monitoring Stations network, operated by the National Institute of Environmental Research (NIER) for 255 urban stations for the years 2014 to 2017 across the Republic of Korea. The network measures and provides real-time air pollutant concentrations such as sulfur dioxide (SO_2_), carbon monoxide (CO), ozone (O_3_), and nitrogen dioxide (NO_2_). Since the CNN model requires continuously measured data for training/testing, we input the missing values of observational datasets. For these missing values, we used SOFT-IMPUTE by Mazumder et al. (2010)^26^. We then extracted the concentrations of air pollutants from CMAQ and meteorological parameters from the WRF (processed by Meteorology-Chemistry Interface Processor (MCIP) modules of the CMAQ model). For this purpose, we used the temporally and spatially matched CMAQ grid points of the NIER station locations. Table S9 (Supplementary Document) displays all of the parameters extracted from the MCIP and CMAQ.

After acquiring hourly meteorological fields from the WRF model, previous day pollutant concentrations from observations and the forecasted parameters from the CMAQ runs, we prepared the input for each station in the form of a two-dimensional matrix in which each column represented a specific parameter (meteorology or gaseous concentration), and each row represented hourly values. Figure S15 (Supplementary Document) represents the schematic diagram of the data preparation used for the 14-day forecasting. For each day (24 h), we prepared separate models in such a way that inputs remained the same for all the models, but the output (target) is changed from day 1, day 2 until day 14. Thus, a total of 14 models were prepared, one for each forecasting day. Then we trained the model for three years (i.e., 2014 to 2016) and evaluated it for the year 2017 (Note: 2017 was never used for training the model; the training of the CNN models was done using data from 2014 to 2016; 1096 days). The input dataset consisted of previous 24-h observed air pollutant concentrations and the meteorology generated by the WRF; and the following 24-h forecasted air-pollutants from the CMAQ model (in total 50 input parameters). The output dataset consisted of the next day 24-h observed ozone concentration for day 1, 24 to 48 h for day 2, 48 to 72 h for day 3, and so on. After we defined the inputs and outputs, we combined the datasets from all stations to construct a matrix for training/testing a generalized deep CNN model across the spatial domain. Since we had 255 stations and three years of hourly data for training, we trained the model with 279,480 (1096 × 255) examples (days), which were further split randomly into equal parts so that the model was trained on one half and validated on the other. Since each parameter had a unique range of values, we normalized each one between zero and 1 to remove the model bias toward any specific high or low valued parameter. It has been observed that having a different maximum and minimum for a training and prediction set destabilizes the model and produces varied results over different runs. Therefore, for the normalization process, we chose “global” maximum and minimum values for each parameter. These global maximum and minimum values guaranteed that none of the hourly values exceeded a certain level; thus, the normalization process remained independent of the temporal and spatial variations. After normalization, we used the deep CNN architecture (defined in the previous section) to train the model and generated two models, each with a unique loss function. Once the model was generated, it was used to predict the entire year of 2017.

For long-term training and prediction, we prepared the dataset so that it had the same inputs, but we changed the outputs from the first day to the second, third, and fourth days and so on until the fourteenth day (Figure S15 in the supplementary document presents a schematic diagram of the data setup used in this study.) Hence, with two loss functions and 14 days of predictions, we had 28 models to evaluate.

## Supplementary Information

Supplementary Information.

## Data Availability

The test/train/validation data are available for non-commercial research purposes by contacting the corresponding author.

## Code availability

The code for the algorithm development, evaluation, and statistical analysis is freely available for non-commercial research purposes by contacting the corresponding author.
