# Supplementary material for: A novel CMAQ-CNN hybrid model to forecast hourly surface-ozone concentrations 14 days in advance
Source: Sci Rep. 2021 May 25;11:10891. doi: 10.1038/s41598-021-90446-6 (PMC8149875; doi:10.1038/s41598-021-90446-6)
Supplement: Supplementary file 1 — Supplementary Information. [file 41598_2021_90446_MOESM1_ESM.docx]

**A Novel CMAQ-CNN Hybrid Model to Forecast Hourly Surface-Ozone Concentrations Fourteen Days in Advance**

Authors: Alqamah Sayeed^a^, Yunsoo Choi^*a^, Ebrahim Eslami^a, b^, Jia Jung^a^, Yannic Lops^a^, Ahmed Khan Salman^a^, Jae-Bum Lee^c^, Hyun-Ju Park^c^, Min-Hyeok Choi^c^

^a^Departmcnt of Earth and Atmospheric Sciences, University of Houston, TX 77004

^b^Houston Advanced Research Center, The Woodlands, TX 77381

^c^National Institute of Environmental Research, Incheon Korea

*corresponding author, ychoi23@central.uh.edu

**--------------------------------------------------------------------------------------------------------------------------------------------------------------------------------------------------------**

**Supplementary Document**

**IOA: General Statistical Analysis**

IOA varies between 0 and 1 and indicates the degree of model prediction error. A value of 1 indicates a perfect match and 0 indicates no agreement at all^1^. The IOA can be defined as

$IOA = 1 - \frac{\sum\left( O_{i} - P_{i} \right)^{2}}{\sum\left( {abs(O}_{i} - \bar{O})+{abs(P}_{i} - \bar{O}) \right)^{2}}$ (1)

where $O_{i}$ and $P_{i}$ represent the observed and predicted values, respectively. $\bar{O}$ is the mean of observed values for the entire observation sample.

**Supplementary Figures:**


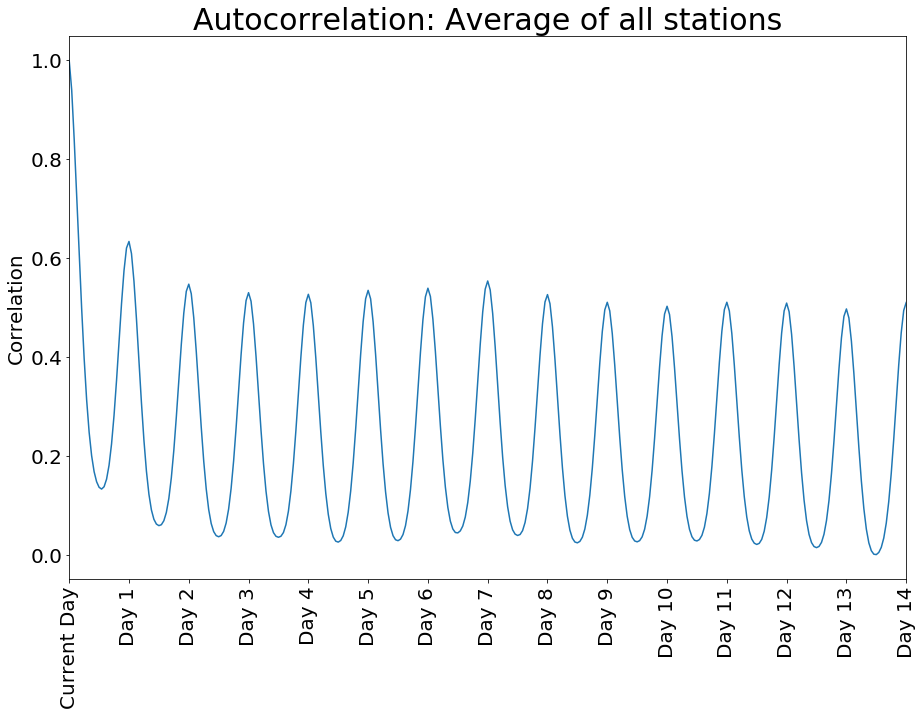


**Figure S1:** Auto-correlations (average of all stations) of the observed current hour ozone concentration with the subsequent hour observed ozone concentration. The X-axis represents the hours, and the y-axis represents the correlation. (Correlation of 0^th^ hour with 0^th^ hour, 1^st^ hour, 2^nd^ hour, and so on. It is analogous to delayed response in electrical signals)


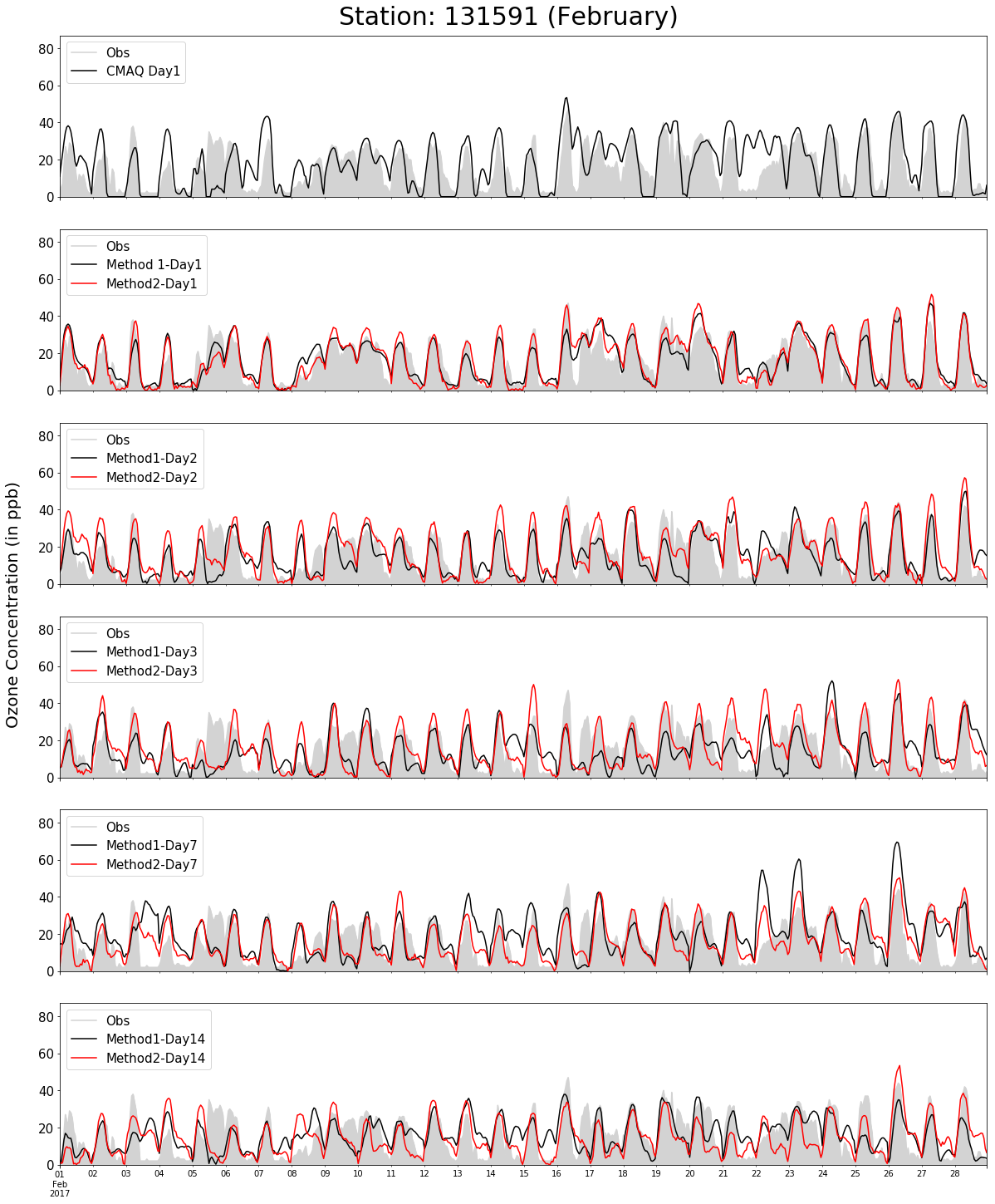


**Figure S2:** Hourly time-series plot for station 131591 for February 2017. The panels from top to bottom show a time-series comparison of the observed ozone concentration with the CMAQ day 1 forecast, the CNN-method 2 day1, day 2, day 3, day 7, and day 14 forecasts, respectively. The X-axis represents the days of the month and the y-axis represents the ozone concentration (in ppb).


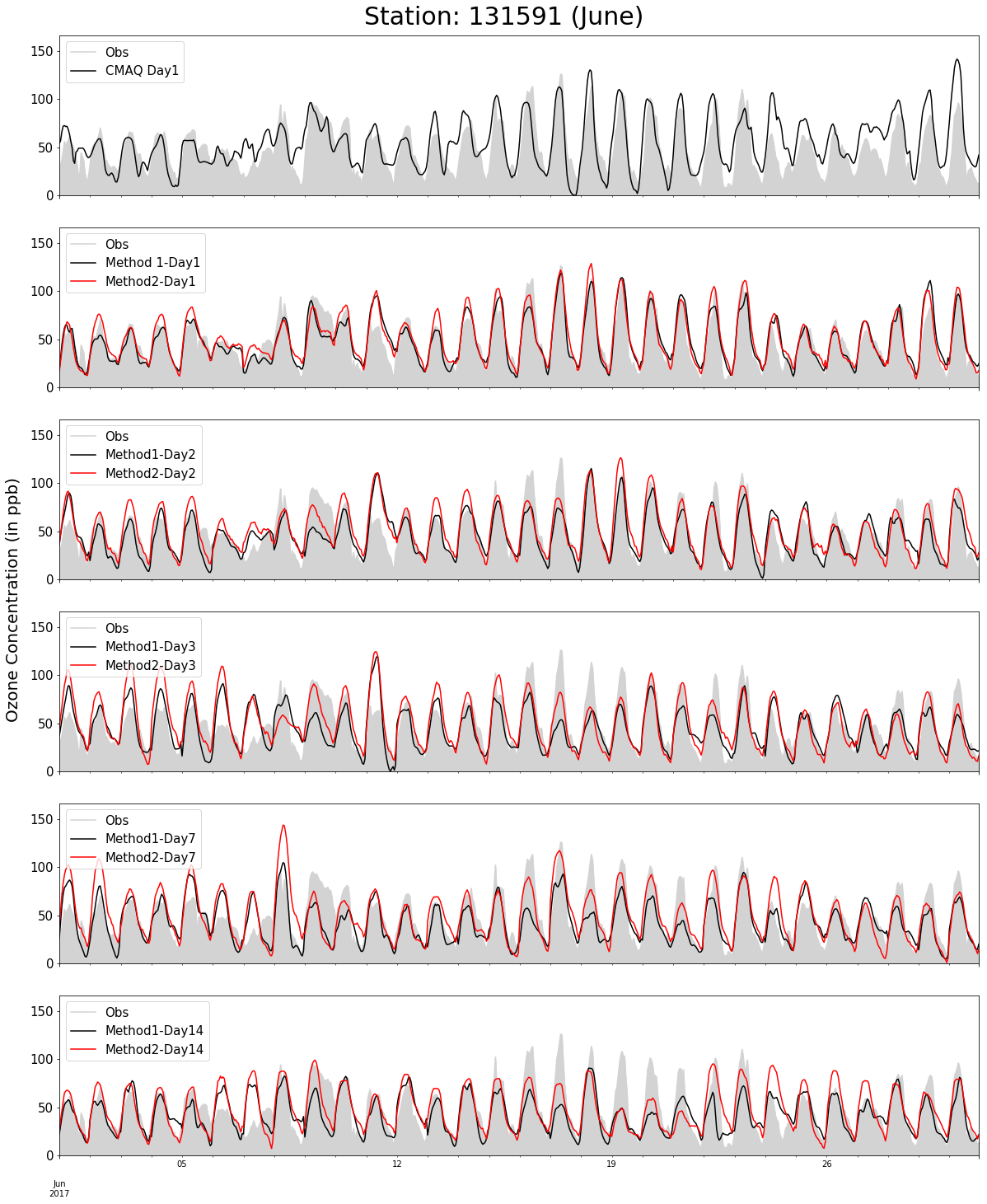


**Figure S3:** Hourly time-series plot for station 131591 for the month of June 2017. The panels from top to bottom show a time-series comparison of the observed ozone concentration with the CMAQ day 1 forecast, the CNN-method 2 day1, day 2, day 3, day 7, and day 14 forecasts, respectively. The X-axis represents the days of the month and the y-axis represents the ozone concentration (in ppb).


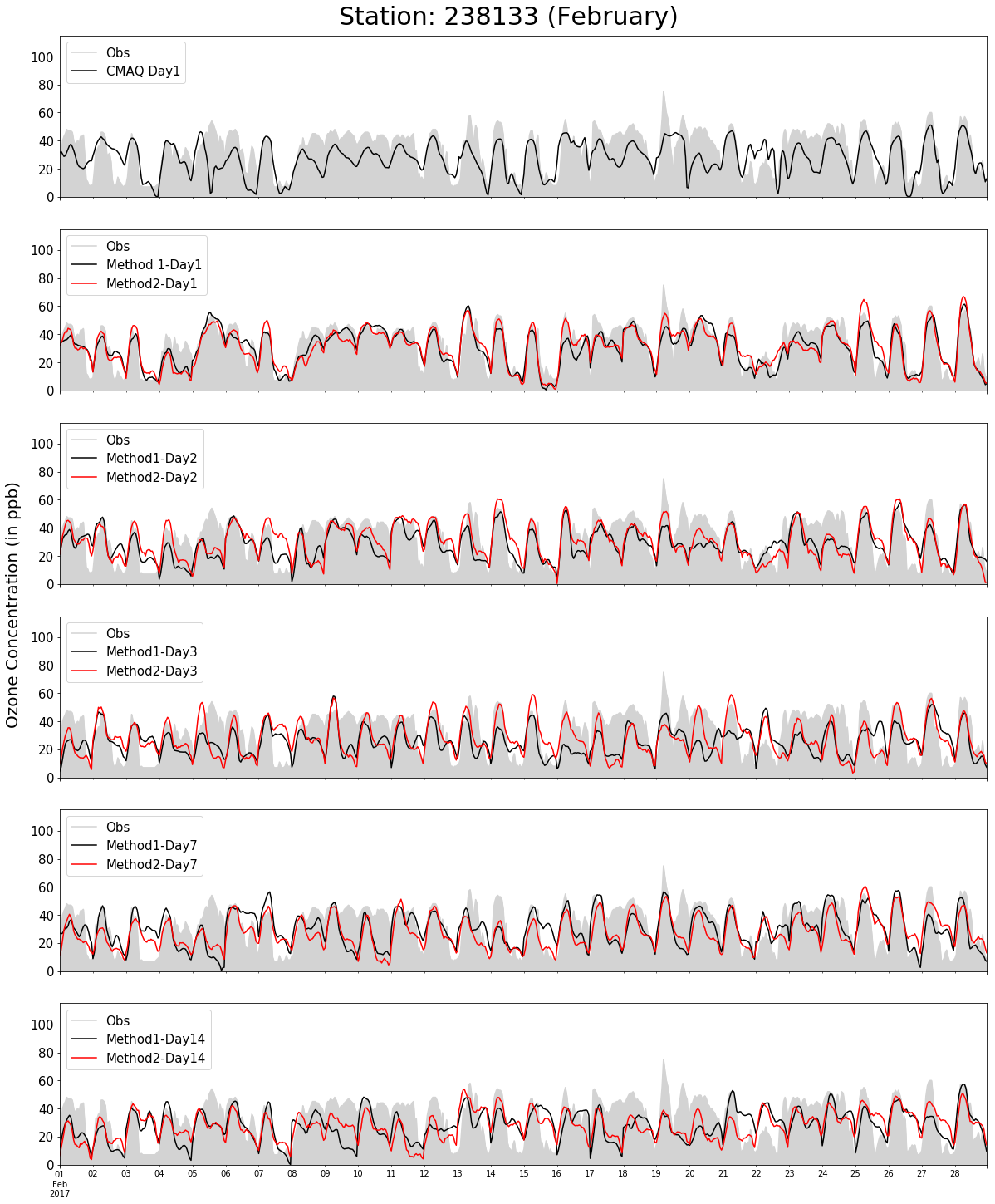


**Figure S4:** Hourly time-series plot for station 238133 for the month of February 2017. The panels from top to bottom show a time-series comparison of the observed ozone concentration with the CMAQ day 1 forecast, the CNN-method 2 day1, day 2, day 3, day 7, and day 14 forecasts, respectively. The X-axis represents the days of the month and the y-axis represents the ozone concentration (in ppb).


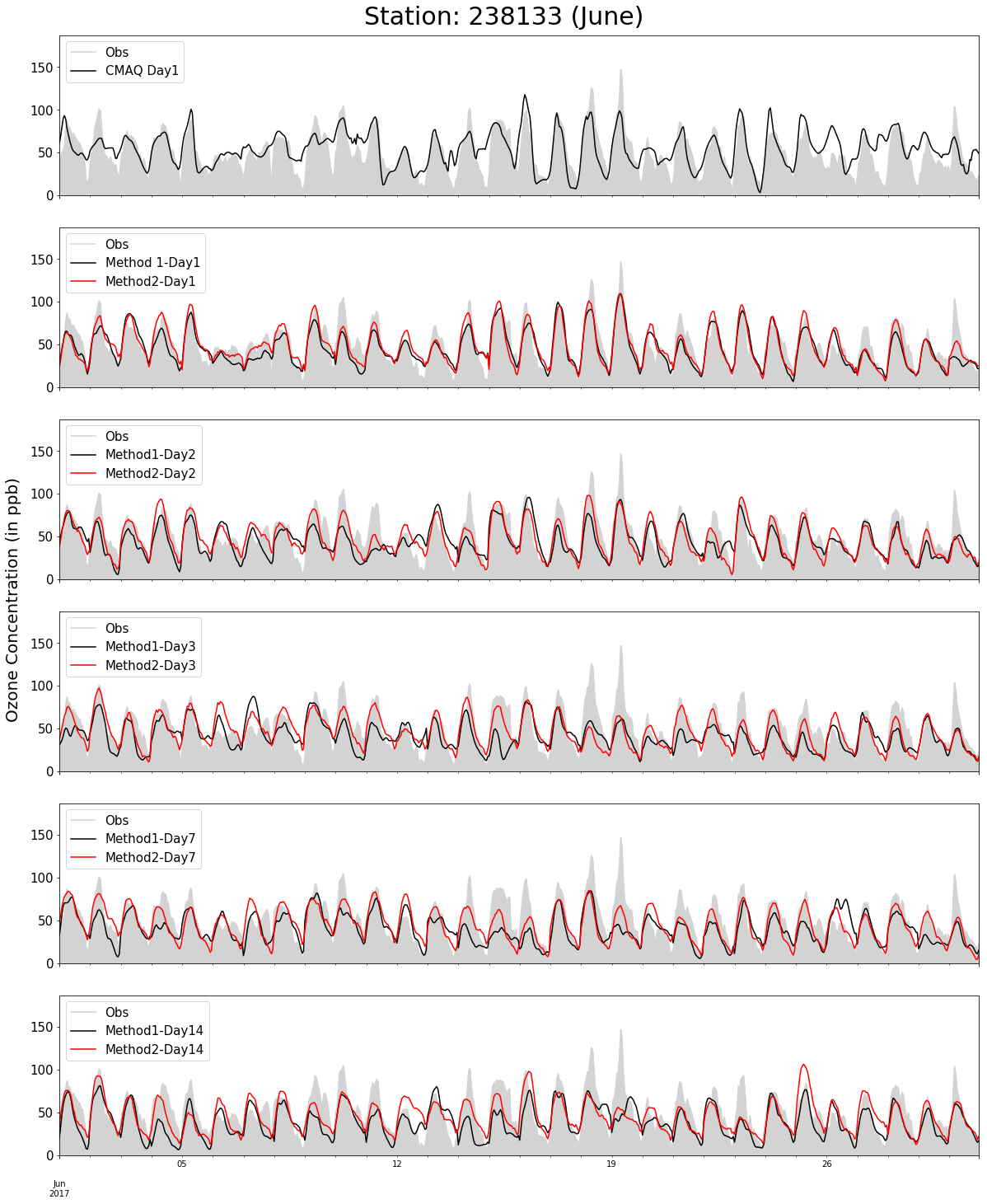


**Figure S5:** Hourly time-series plot for station 238133 for the month of June 2017. The panels from top to bottom show a time-series comparison of the observed ozone concentration with the CMAQ day 1 forecast, the CNN-method 2 day1, day 2, day 3, day 7, and day 14 forecasts, respectively. The X-axis represents the days of the month and the y-axis represents the ozone concentration (in ppb).


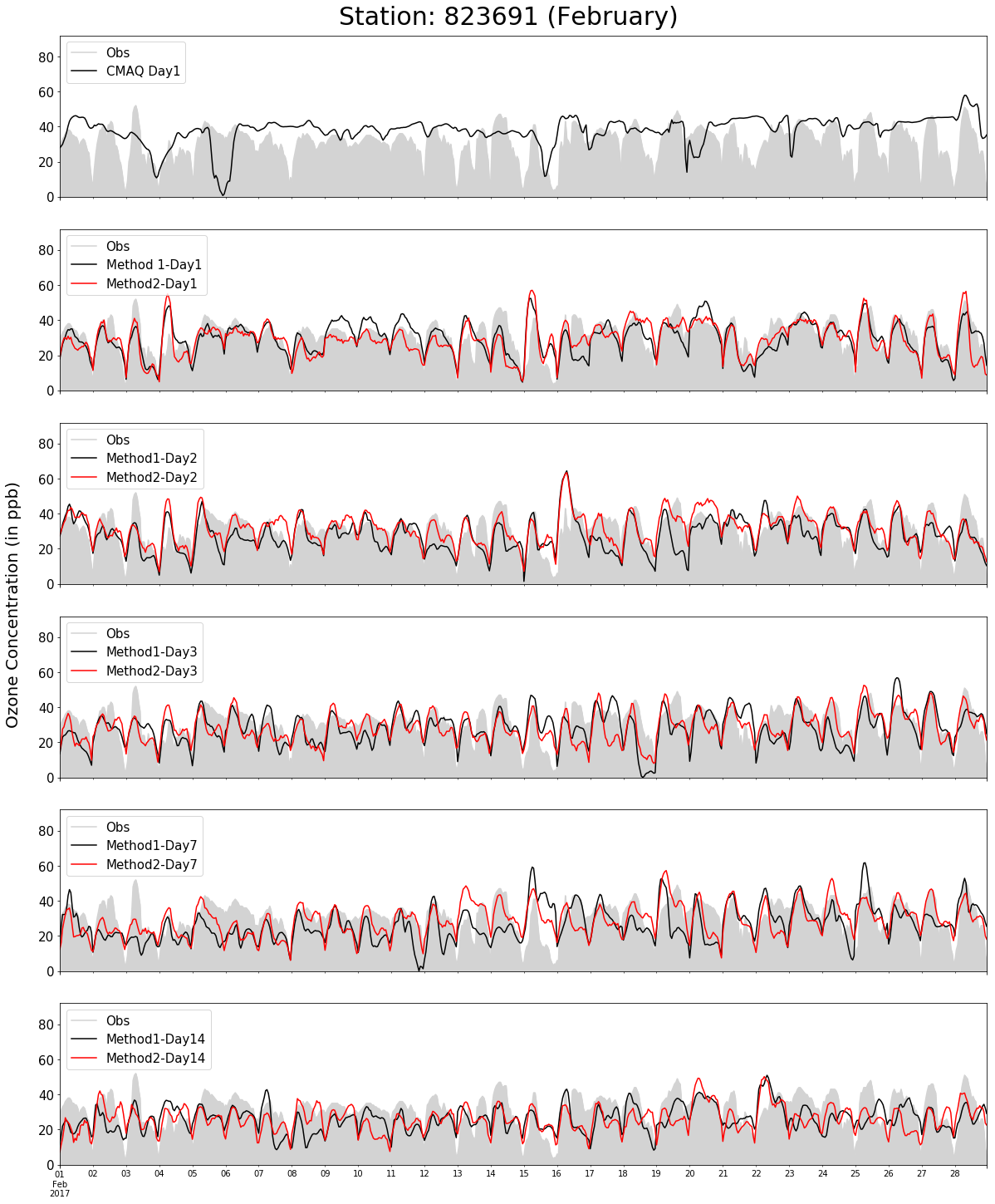


**Figure S6:** Hourly time-series plot for station 823691 for the month of February 2017. The panels from top to bottom show a time-series comparison of the observed ozone concentration with the CMAQ day 1 forecast, the CNN-method 2 day1, day 2, day 3, day 7, and day 14 forecasts, respectively. The X-axis represents the days of the month and the y-axis represents the ozone concentration (in ppb).


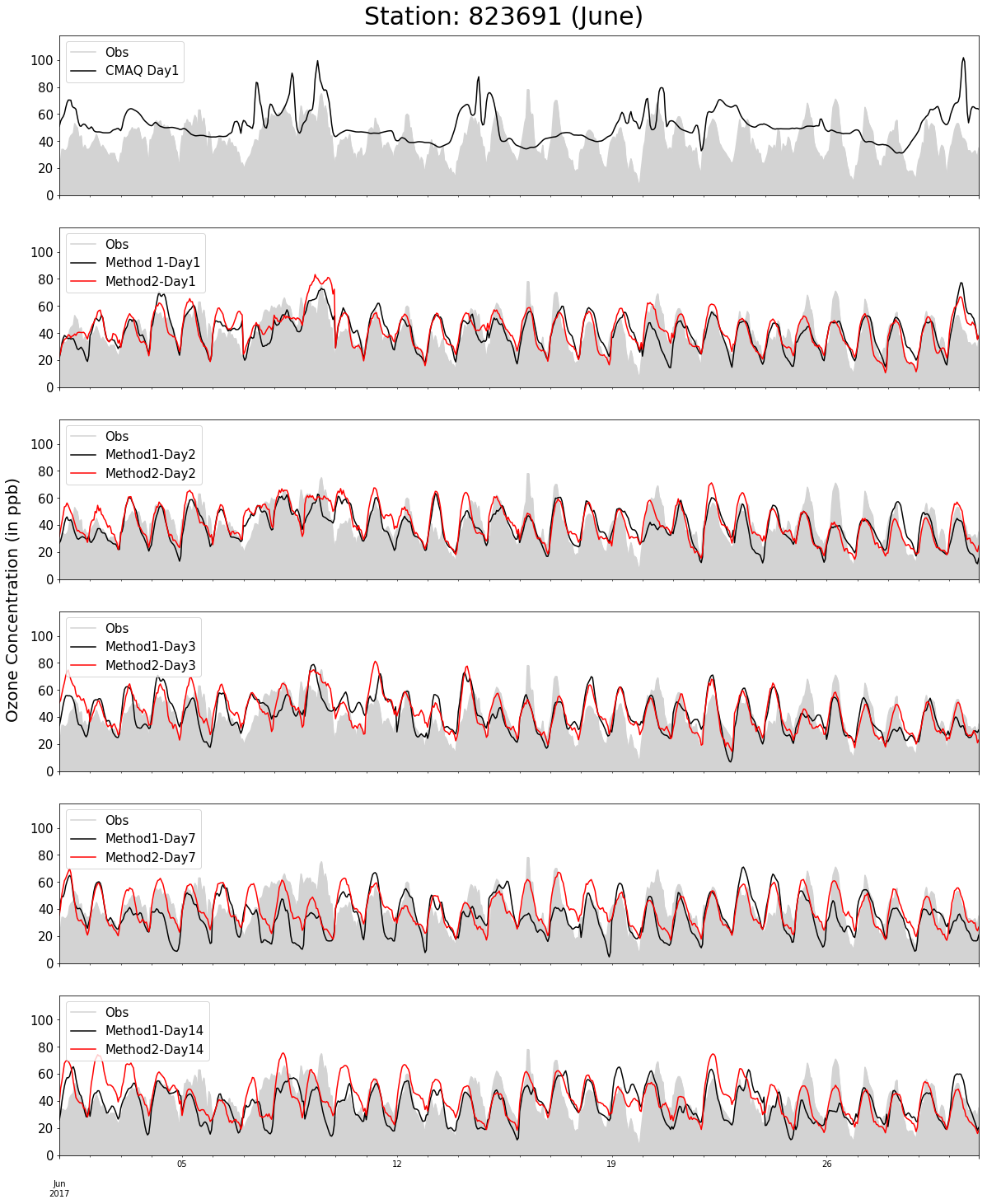


**Figure S7:** Hourly time-series plot for station 823691 for the month of June 2017. The panels from top to bottom show a time-series comparison of the observed ozone concentration with the CMAQ day 1 forecast, the CNN-method 2 day1, day 2, day 3, day 7, and day 14 forecasts, respectively. The X-axis represents the days of the month and the y-axis represents the ozone concentration (in ppb).


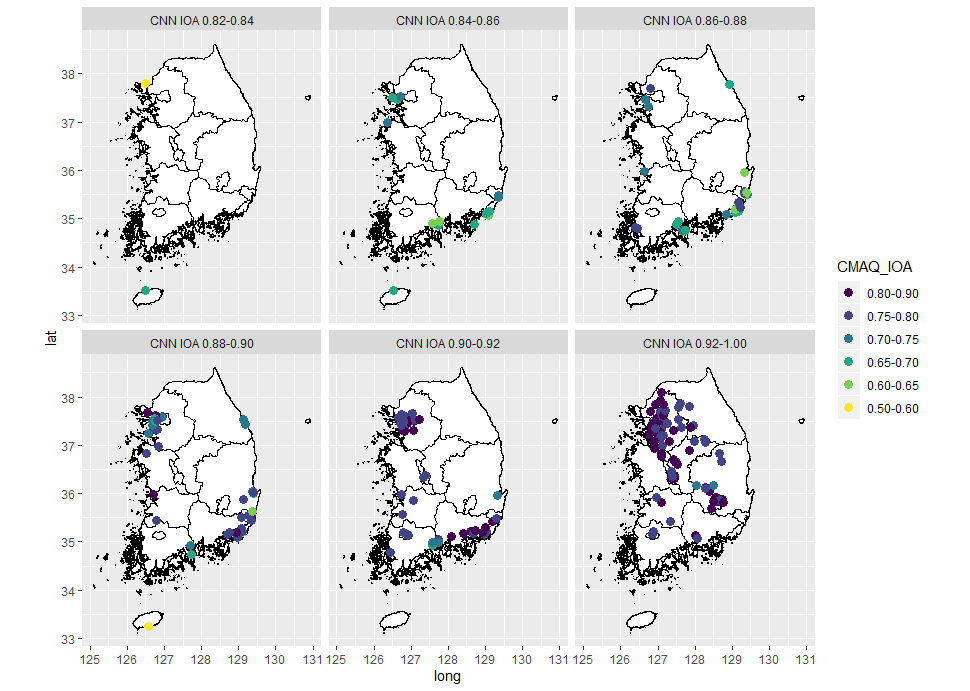


**Figure S8:** Station-based CNN-IOA binned in specific ranges. A colored dot represents the location of the station, and a specific color represents the CMAQ-IOA. (Figures are created using R ggplot2 ^3^: <https://ggplot2.tidyverse.org/>)


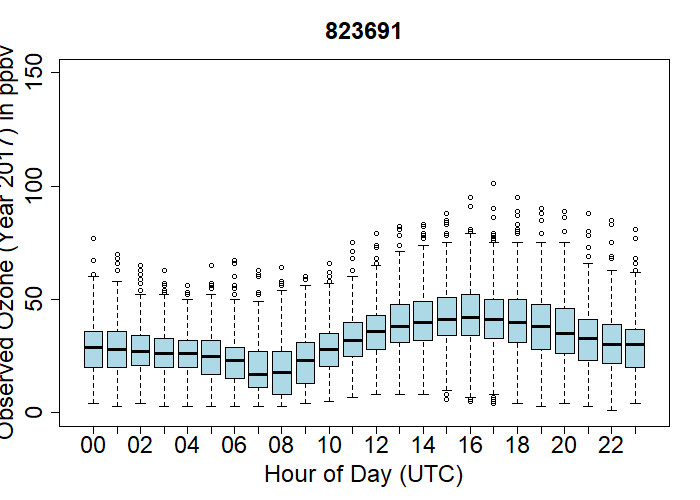

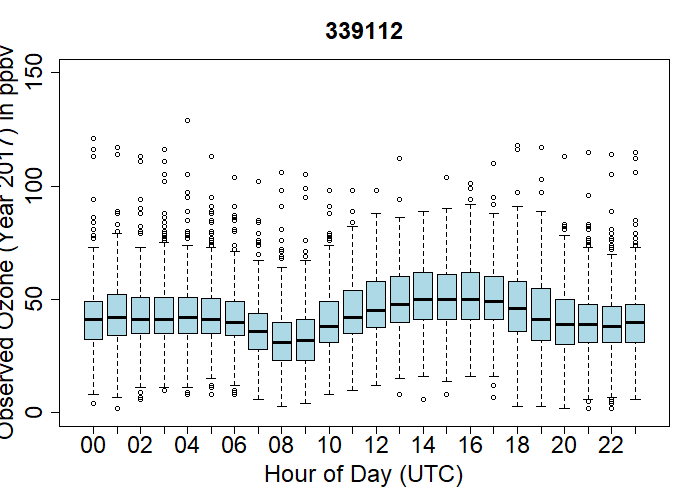

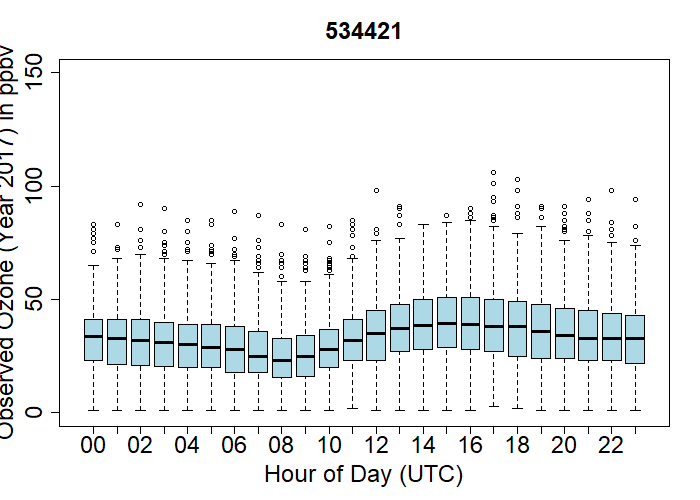

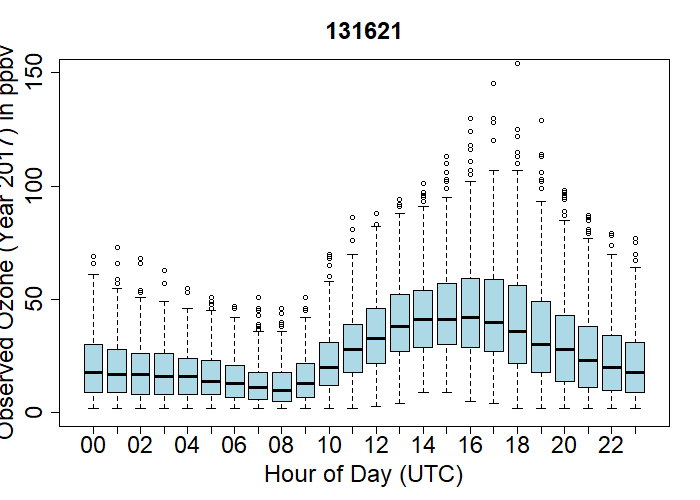

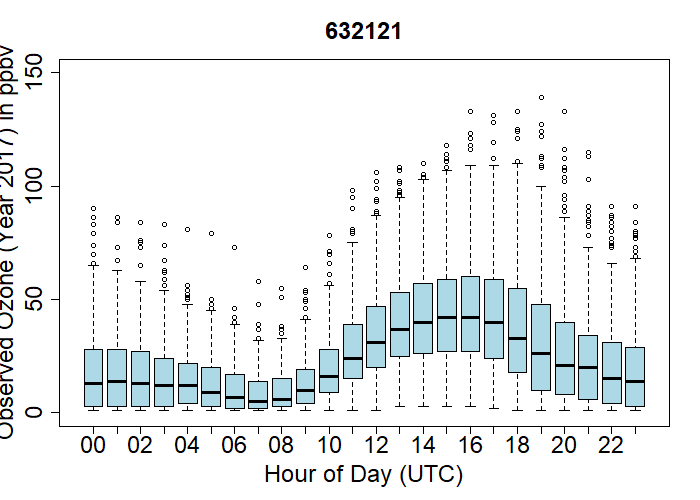

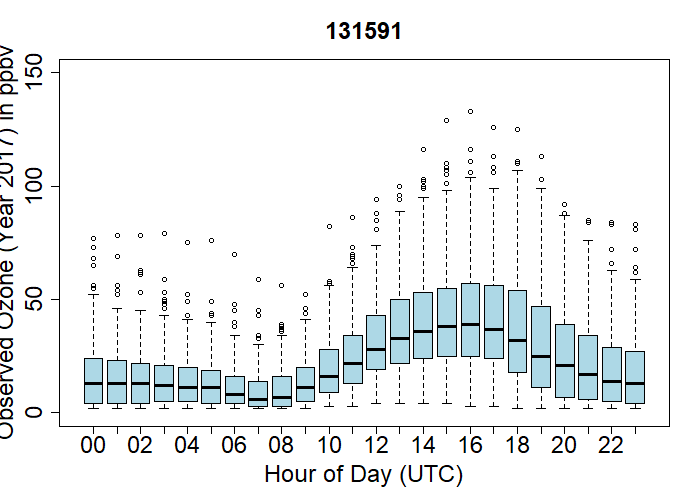


(f)

(e)

(d)

(c)

(b)

(a)

**Figure S9:** Box and whisker plot 24-hour observed ozone concentration throughout the year 2017. a, b and c are the three worst-performing stations. d, e, and f are the best performing station.


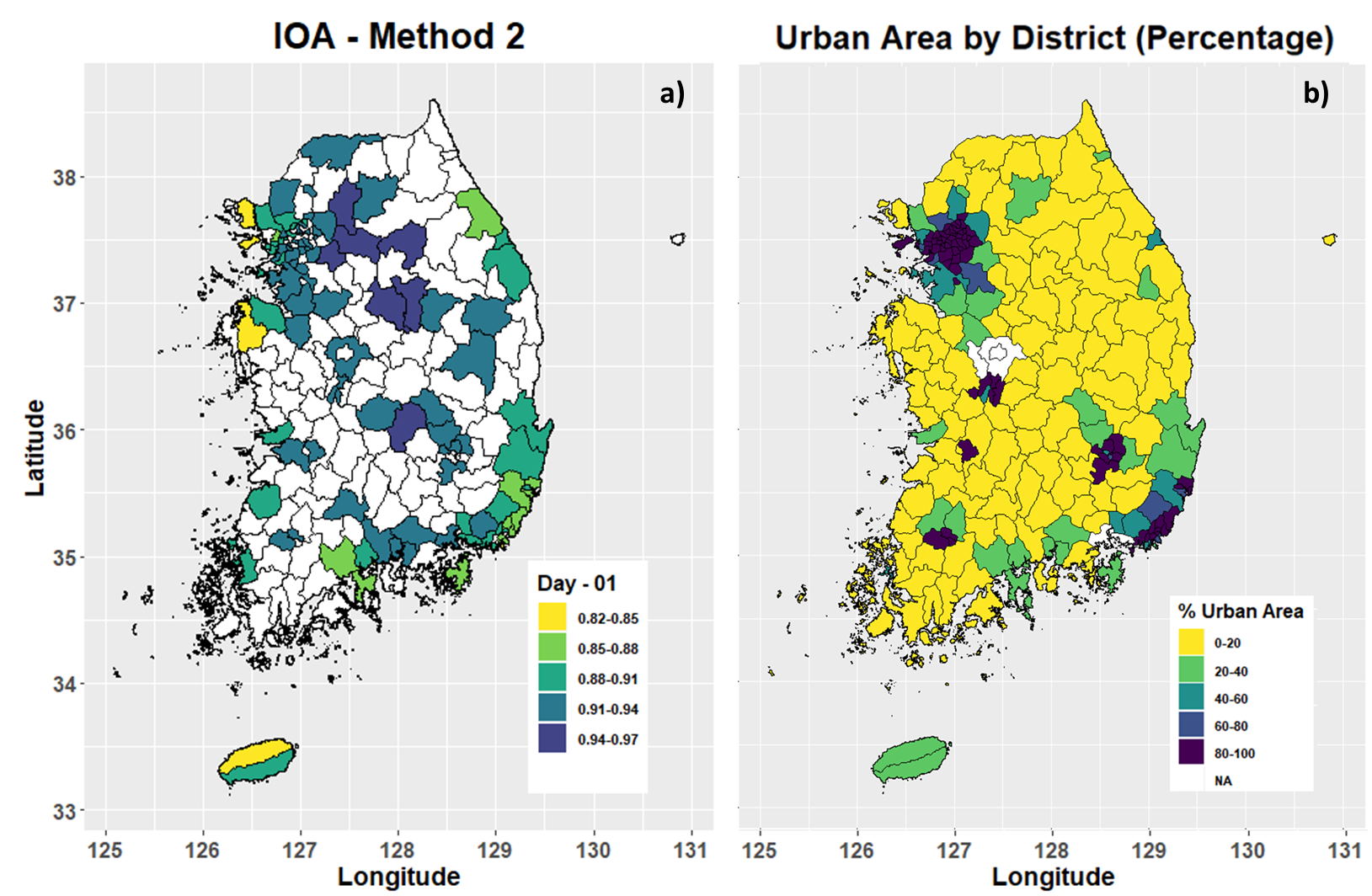


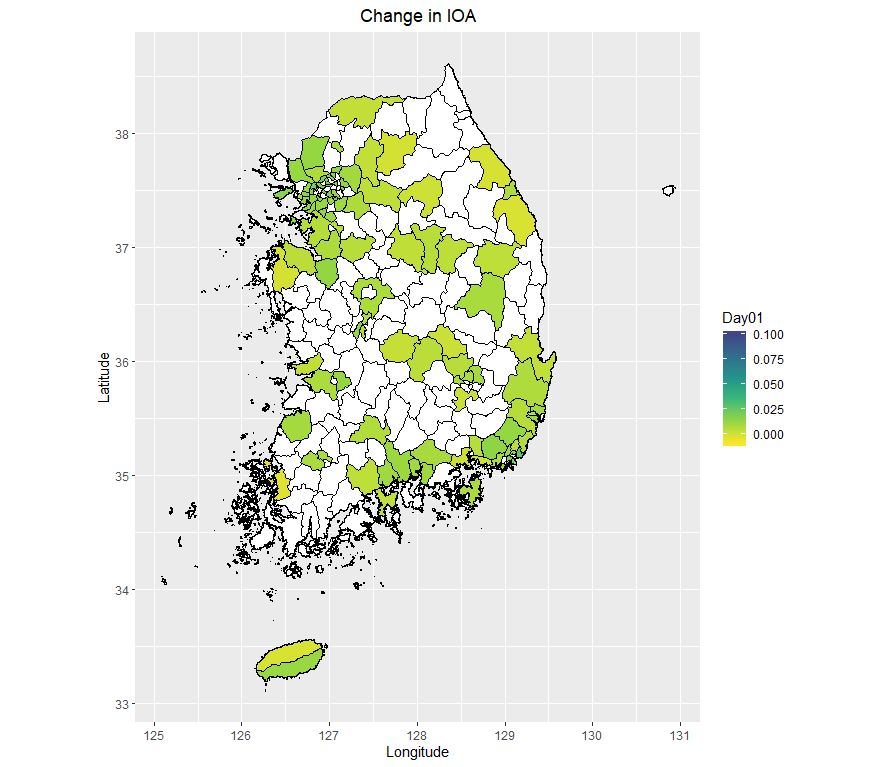
**Figure S10:** a) District-wise IOA based on Method 2 of CNN. b) Percentage of Urban area^4^ in each district of Korea. (Figures are created using R ggplot2 ^3^: <https://ggplot2.tidyverse.org/>)


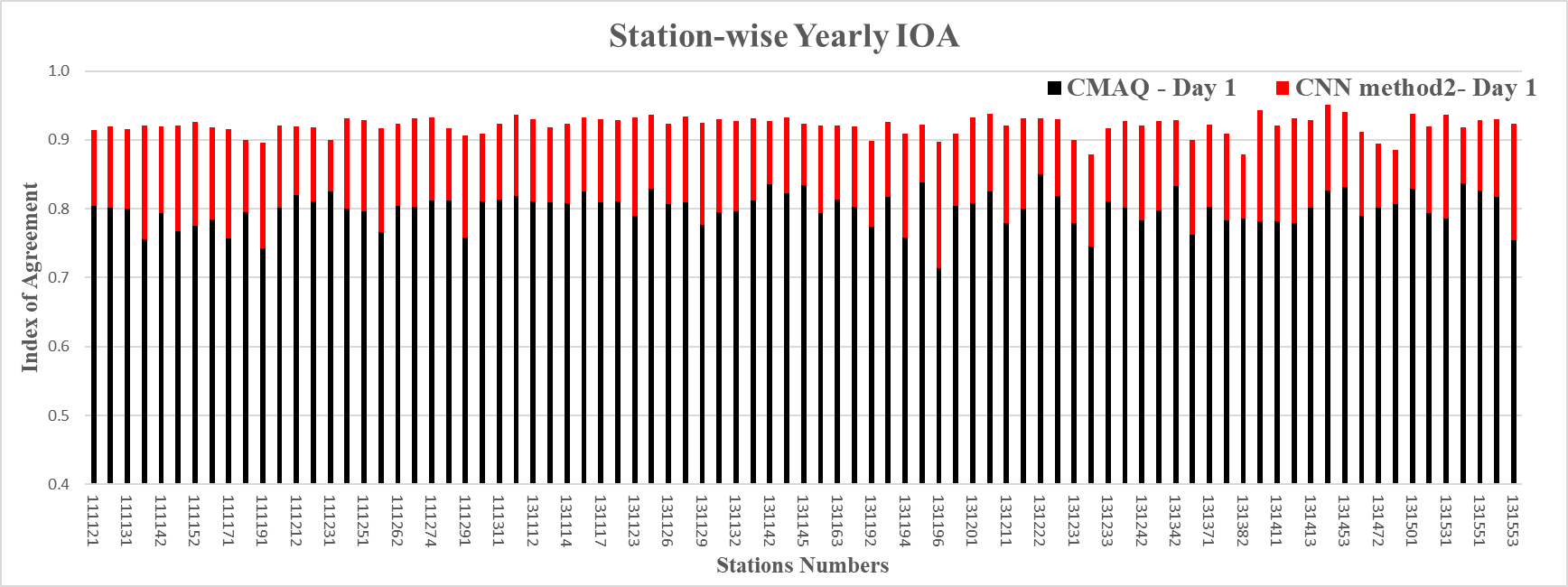


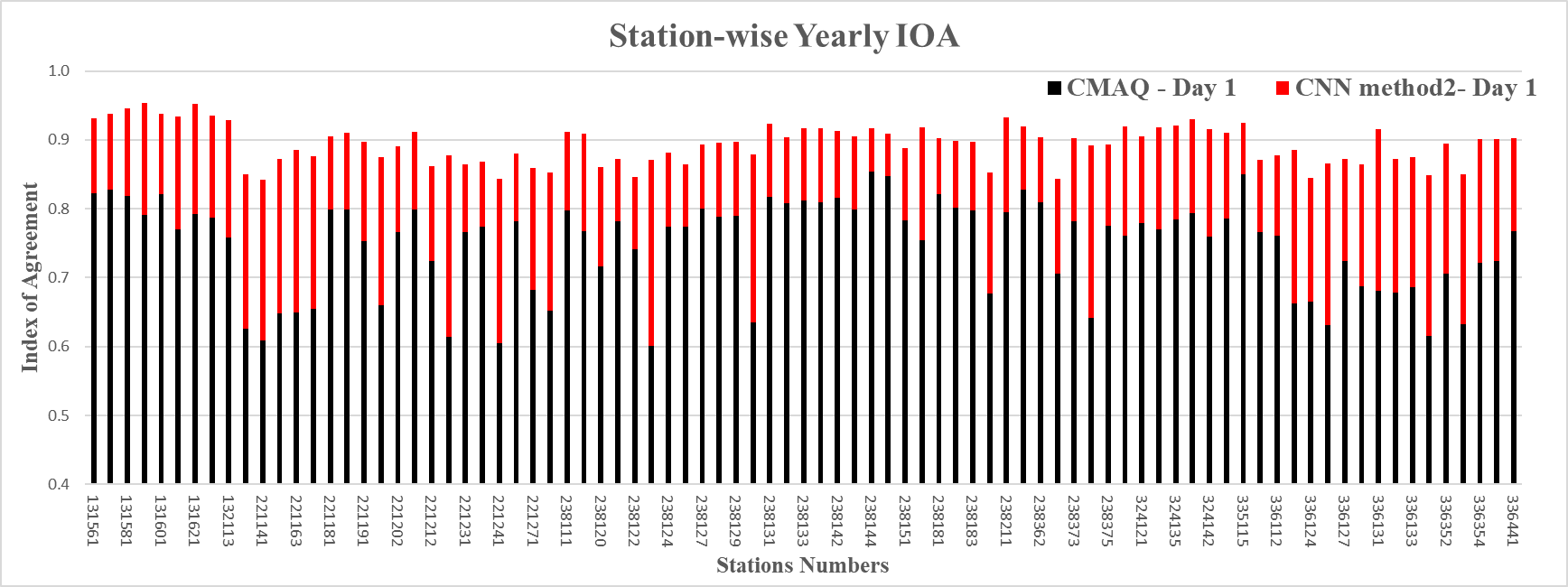


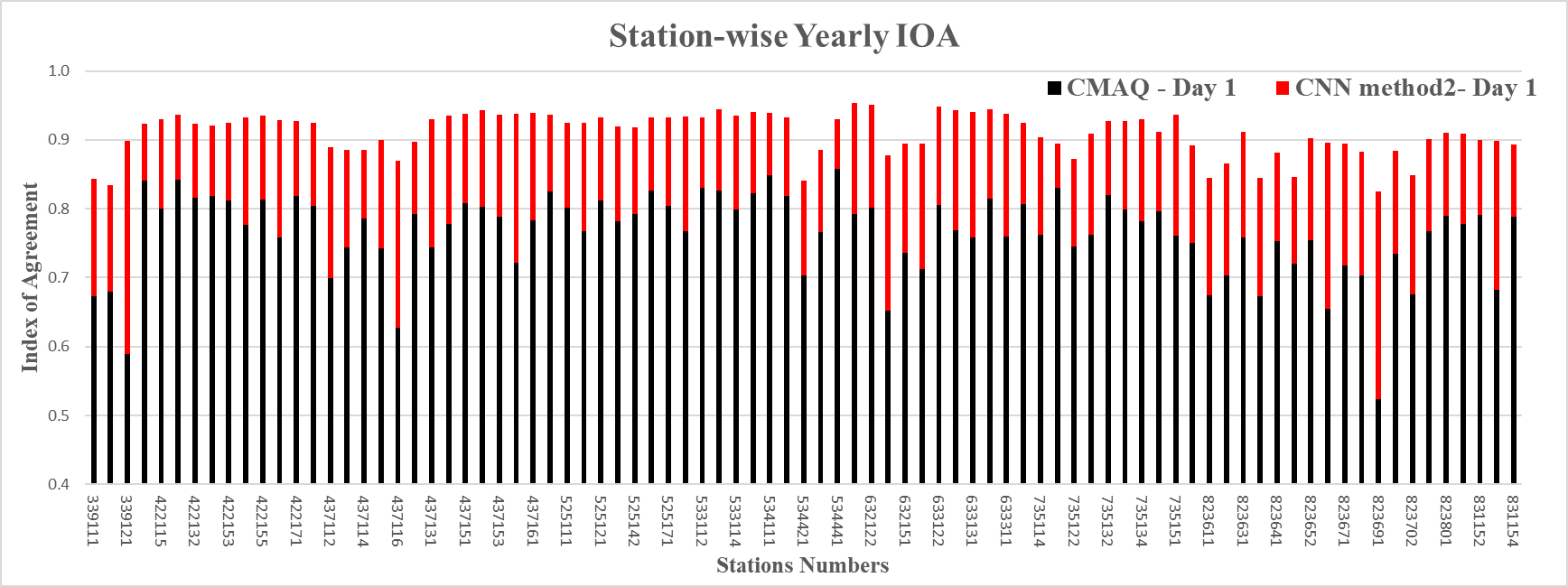


**Figure S11:** Station-wise yearly index of agreement (IOA) for the CMAQ and the CNN-method 2 model for the day one forecast. The black bar represents the CMAQ models IOA. The sum of the black bar and red bar represents the IOA for the CNN-method 2 model. The red bar individually represents the absolute increase in the IOA from the CMAQ model. The X-axis represents IOA and the y-axis represents the station number.


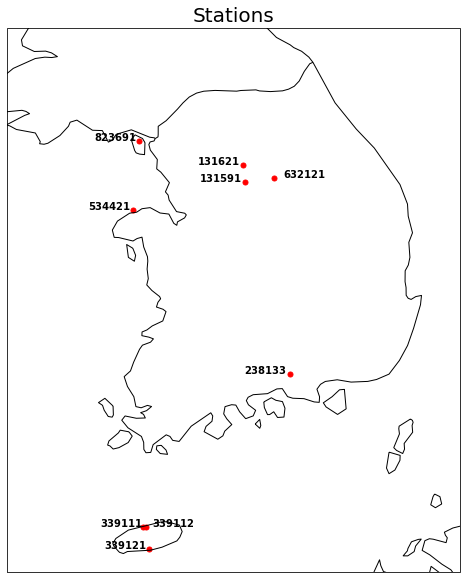


**Figure S12:** Location of few stations specifically mentioned in the study. (Figures are created using python cartopy package^5^: https://scitools.org.uk/cartopy/docs/latest/)


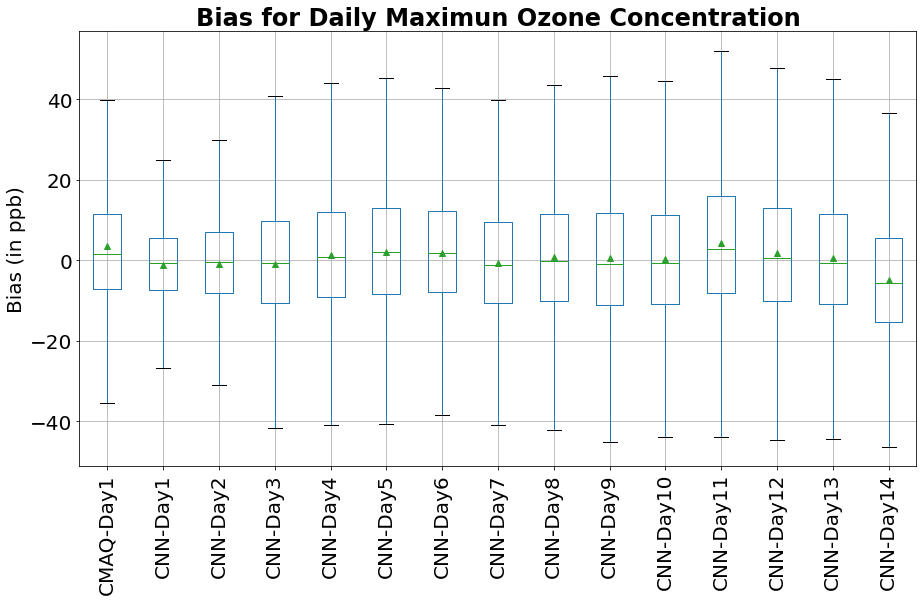


**Figure S13:** Box plot of bias of daily maximum all stations combined. The x-axis represents the prediction days, and the y-axis represents the bias in ppb. The green line represents the median of bias, and the green triangle in each box represents the mean bias for that model—the extent of the box represents the interquartile range (IQR), i.e., 25^th^ to 75^th^ percentile value.

**
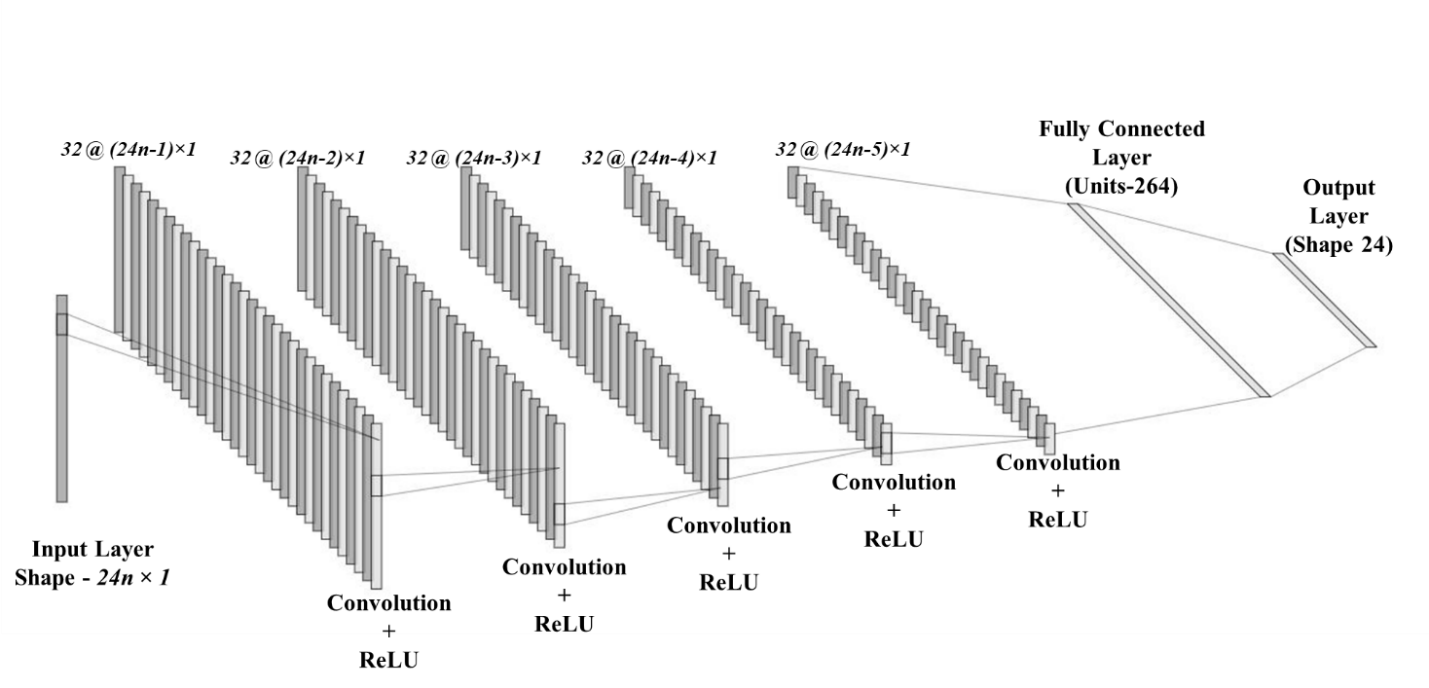
**

**Figure S14:** Schematic diagram of the Convolution Neural Network. ‘n’ is the number of input parameters (meteorology, air quality, and observations) used. Here, n=50 (32 meteorology, 14 air-quality parameters and 4 previous day observations), so there are 1200 (=52 × 24) inputs and 24 outputs. Schematics are prepared by the NNSVG tool.^2^


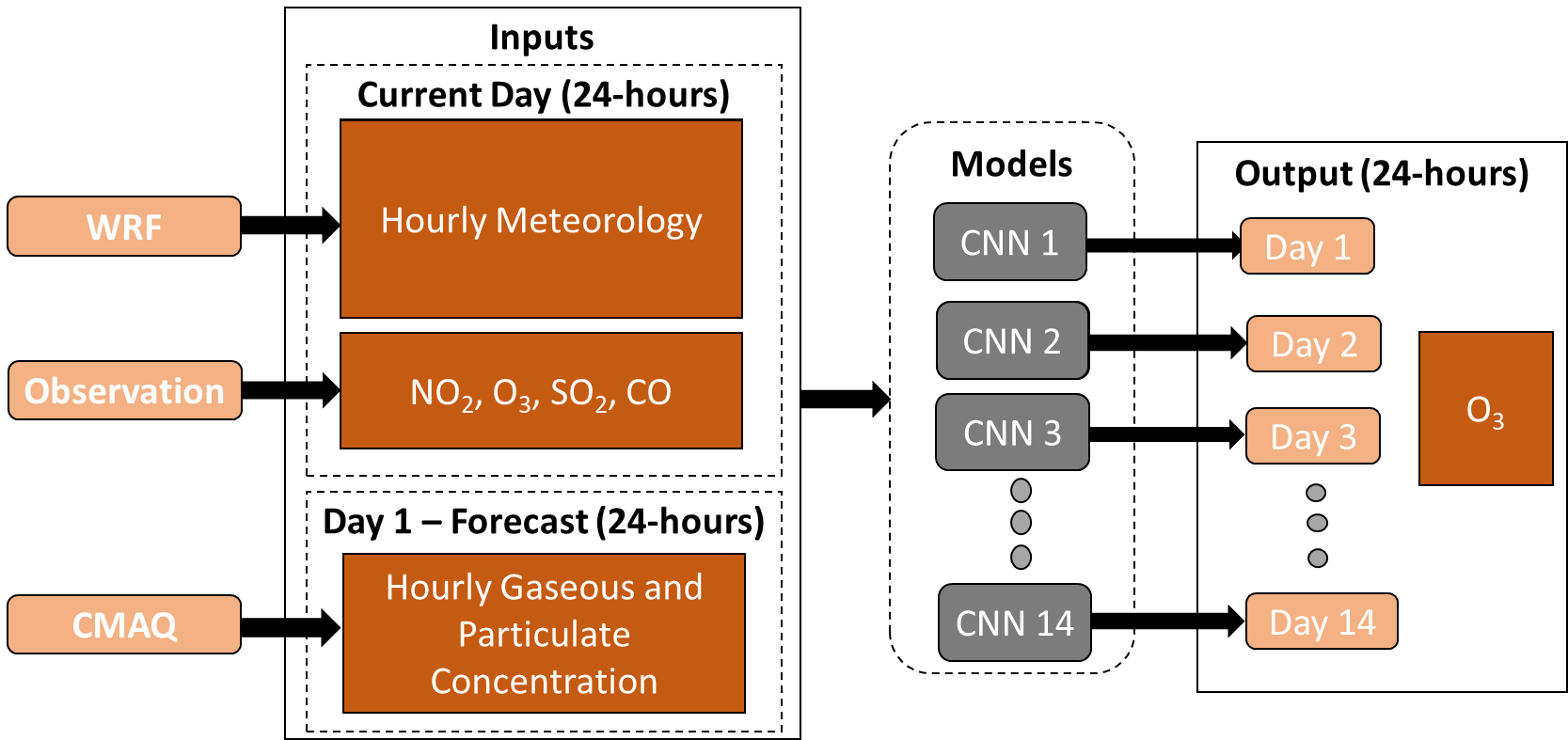


**Figure S15:** Schematic diagram of the process flow of the CNN model. Note: there are 1200 input parameters (32 meteorology, 14 parameters from CMAQ and 4 parameters from previous day observation; 50 × 24 = 1200) for 24 output parameters (1 day; 24-hour ozone concentration) for each day model.

**Supplementary Table:**

**Table S1: Average of all stations mean bias for the CMAQ model and both methods of CNN model**

| **Mean Bias** | **Day 1** | **Day 2** | **Day 3** | **Day 4** | **Day 5** | **Day 6** | **Day 7** | **Day 8** | **Day 9** | **Day 10** | **Day 11** | **Day 12** | **Day 13** | **Day 14** |
| --- | --- | --- | --- | --- | --- | --- | --- | --- | --- | --- | --- | --- | --- | --- |
| **CMAQ** | 1.21 | NA | NA | NA | NA | NA | NA | NA | NA | NA | NA | NA | NA | NA |
| **Method1/ MSE** | -1.23 | -2.11 | -3.23 | -2.59 | -1.55 | -2.06 | -1.62 | -1.48 | -2.08 | -2.78 | -2.26 | -2.47 | -3.24 | -2.62 |
| **Method2/ IOA** | -0.96 | -1.01 | -1.69 | -0.22 | -0.25 | 0.65 | -0.84 | -0.54 | -0.95 | 0.01 | -0.15 | 0.84 | -0.29 | -1.89 |

**Table S2: Average of all stations root mean squared error for the CMAQ model and both methods of CNN model**

| **RMSE** | **Day 1** | **Day 2** | **Day 3** | **Day 4** | **Day 5** | **Day 6** | **Day 7** | **Day 8** | **Day 9** | **Day 10** | **Day 11** | **Day 12** | **Day 13** | **Day 14** |
| --- | --- | --- | --- | --- | --- | --- | --- | --- | --- | --- | --- | --- | --- | --- |
| **CMAQ** | 18.98 | NA | NA | NA | NA | NA | NA | NA | NA | NA | NA | NA | NA | NA |
| **Method1/ MSE** | 11.00 | 13.48 | 15.82 | 16.14 | 16.05 | 16.00 | 16.03 | 15.80 | 16.43 | 16.61 | 16.55 | 16.39 | 16.38 | 16.45 |
| **Method2/ IOA** | 11.01 | 13.30 | 15.59 | 16.27 | 16.44 | 15.95 | 15.60 | 16.08 | 16.78 | 16.77 | 17.31 | 16.55 | 16.64 | 16.06 |

**Table S3: Average of all stations correlations for the CMAQ model and both methods of CNN model**

| **Correlation** | **Day 1** | **Day 2** | **Day 3** | **Day 4** | **Day 5** | **Day 6** | **Day 7** | **Day 8** | **Day 9** | **Day 10** | **Day 11** | **Day 12** | **Day 13** | **Day 14** |
| --- | --- | --- | --- | --- | --- | --- | --- | --- | --- | --- | --- | --- | --- | --- |
| **CMAQ** | 0.63 | NA | NA | NA | NA | NA | NA | NA | NA | NA | NA | NA | NA | NA |
| **Method1/ MSE** | 0.82 | 0.72 | 0.62 | 0.60 | 0.60 | 0.61 | 0.60 | 0.61 | 0.59 | 0.58 | 0.59 | 0.59 | 0.59 | 0.59 |
| **Method2/ IOA** | 0.84 | 0.76 | 0.67 | 0.66 | 0.66 | 0.66 | 0.66 | 0.66 | 0.64 | 0.63 | 0.65 | 0.64 | 0.64 | 0.63 |

**Table S4: Average of all stations hit rate for the CMAQ model and both methods of CNN model**

| **Hit Rate** | **Day 1** | **Day 2** | **Day 3** | **Day 4** | **Day 5** | **Day 6** | **Day 7** | **Day 8** | **Day 9** | **Day 10** | **Day 11** | **Day 12** | **Day 13** | **Day 14** |
| --- | --- | --- | --- | --- | --- | --- | --- | --- | --- | --- | --- | --- | --- | --- |
| **CMAQ** | 0.77 | NA | NA | NA | NA | NA | NA | NA | NA | NA | NA | NA | NA | NA |
| **Method1/ MSE** | 0.67 | 0.50 | 0.39 | 0.36 | 0.40 | 0.42 | 0.41 | 0.37 | 0.38 | 0.34 | 0.37 | 0.33 | 0.31 | 0.34 |
| **Method2/ IOA** | 0.80 | 0.74 | 0.58 | 0.66 | 0.63 | 0.64 | 0.57 | 0.62 | 0.61 | 0.60 | 0.69 | 0.61 | 0.63 | 0.47 |

**Table S5: Average of all stations false alarm rate for the CMAQ model and both methods of CNN model**

| **False Alarm Rate** | **Day 1** | **Day 2** | **Day 3** | **Day 4** | **Day 5** | **Day 6** | **Day 7** | **Day 8** | **Day 9** | **Day 10** | **Day 11** | **Day 12** | **Day 13** | **Day 14** |
| --- | --- | --- | --- | --- | --- | --- | --- | --- | --- | --- | --- | --- | --- | --- |
| **CMAQ** | 0.43 | NA | NA | NA | NA | NA | NA | NA | NA | NA | NA | NA | NA | NA |
| **Method1/ MSE** | 0.23 | 0.28 | 0.44 | 0.47 | 0.49 | 0.48 | 0.46 | 0.47 | 0.49 | 0.51 | 0.53 | 0.50 | 0.48 | 0.47 |
| **Method2/ IOA** | 0.28 | 0.35 | 0.48 | 0.50 | 0.50 | 0.49 | 0.47 | 0.48 | 0.52 | 0.53 | 0.54 | 0.53 | 0.51 | 0.49 |

**Table S6: Average of all stations critical success index for the CMAQ model and both methods of CNN model**

| **Critical Success Index** | **Day 1** | **Day 2** | **Day 3** | **Day 4** | **Day 5** | **Day 6** | **Day 7** | **Day 8** | **Day 9** | **Day 10** | **Day 11** | **Day 12** | **Day 13** | **Day 14** |
| --- | --- | --- | --- | --- | --- | --- | --- | --- | --- | --- | --- | --- | --- | --- |
| **CMAQ** | 0.47 | NA | NA | NA | NA | NA | NA | NA | NA | NA | NA | NA | NA | NA |
| **Method1/ MSE** | 0.56 | 0.43 | 0.30 | 0.28 | 0.30 | 0.31 | 0.31 | 0.29 | 0.29 | 0.25 | 0.27 | 0.25 | 0.24 | 0.26 |
| **Method2/ IOA** | 0.61 | 0.53 | 0.38 | 0.40 | 0.39 | 0.40 | 0.38 | 0.39 | 0.37 | 0.36 | 0.38 | 0.36 | 0.38 | 0.32 |

**Table S7: Average of all stations equitable threat score for the CMAQ model and both methods of CNN model**

| **Equitable Thread Score** | **Day 1** | **Day 2** | **Day 3** | **Day 4** | **Day 5** | **Day 6** | **Day 7** | **Day 8** | **Day 9** | **Day 10** | **Day 11** | **Day 12** | **Day 13** | **Day 14** |
| --- | --- | --- | --- | --- | --- | --- | --- | --- | --- | --- | --- | --- | --- | --- |
| **CMAQ** | 0.34 | NA | NA | NA | NA | NA | NA | NA | NA | NA | NA | NA | NA | NA |
| **Method1/ MSE** | 0.47 | 0.34 | 0.20 | 0.18 | 0.19 | 0.20 | 0.20 | 0.18 | 0.18 | 0.15 | 0.15 | 0.15 | 0.15 | 0.16 |
| **Method2/ IOA** | 0.51 | 0.42 | 0.26 | 0.26 | 0.25 | 0.26 | 0.25 | 0.26 | 0.23 | 0.21 | 0.22 | 0.21 | 0.24 | 0.20 |

**Table S8: Average of all stations proportion of correct for the CMAQ model and both methods of CNN model**

| **Proportion of Correct** | **Day 1** | **Day 2** | **Day 3** | **Day 4** | **Day 5** | **Day 6** | **Day 7** | **Day 8** | **Day 9** | **Day 10** | **Day 11** | **Day 12** | **Day 13** | **Day 14** |
| --- | --- | --- | --- | --- | --- | --- | --- | --- | --- | --- | --- | --- | --- | --- |
| **CMAQ** | 0.80 | NA | NA | NA | NA | NA | NA | NA | NA | NA | NA | NA | NA | NA |
| **Method1/ MSE** | 0.88 | 0.84 | 0.79 | 0.78 | 0.78 | 0.78 | 0.78 | 0.78 | 0.77 | 0.76 | 0.76 | 0.77 | 0.77 | 0.77 |
| **Method2/ IOA** | 0.88 | 0.85 | 0.78 | 0.77 | 0.76 | 0.77 | 0.78 | 0.78 | 0.75 | 0.74 | 0.73 | 0.74 | 0.76 | 0.77 |

**Table S9:** List of parameters from WRF/MCIP and CMAQ used to train the CNN model.

| **Abb.** | **Variable Name (WRF/MCIP)** | **Units** |
| --- | --- | --- |
| **PRSFC** | Surface Pressure | Pascal |
| **USTAR** | Cell Averaged Friction Velocity | m/s |
| **WSTAR** | Convective Velocity Scale | m/s |
| **PBL** | Planetary Boundary Level Height | M |
| **MOLI** | Inverse Of Monin-Onukhov Length | 1/m |
| **HFX** | Sensible Heat Flux | watt/m^2^ |
| **RADYNI** | Inverse Of Aerodynamic Resistance | m/s |
| **RSTOMI** | Inverse Of Bulk Stomatal Resistance | m/s |
| **TEMPG** | Skin Temperature At Ground | Kelvin |
| **TEMP2** | Temperature At 2 M | Kelvin |
| **Q2** | Mixing Ratio At 2 M | Kg/Kg |
| **WSPD10** | Wind Speed At 10 M | m/s |
| **WDIR10** | Wind Direction At 10 M | Degrees |
| **GLW** | Longwave Radiation At Ground | watt/m^2^ |
| **GSW** | Solar Radiation Absorbed At Ground | watt/m^2^ |
| **RGRND** | Solar Rad Reaching Sfc | watt/m^3^ |
| **RN** | Nonconvec. Pcpn Per Met Tstep | cm |
| **RC** | Convective Pcpn Per Met TSTEP | cm |
| **CFRAC** | Total Cloud Fraction | fraction |
| **CLDT** | Cloud Top Layer Height (M) | meter |
| **CLDB** | Cloud Bottom Layer Height (M) | meter |
| **WBAR** | Avg. Liquid Water Content Of Cloud | g/m^3^ |
| **SNOCOV** | Snow Cover | fraction |
| **VEG** | Vegetation Coverage (Decimal) | Fraction |
| **LAI** | Leaf-Area Index | m^2^/m^2^ |
| **SEAICE** | Sea Ice | Fraction |
| **WR** | Canopy Moisture Content | M |
| **SOIM1** | Volumetric Soil Moisture In Top Cm | m^3^/m^3^ |
| **SOIM2** | Volumetric Soil Moisture In Top M | m^3^/m^4^ |
| **SOIT1** | Soil Temperature In Top Cm | Kelvin |
| **SOIT2** | Soil Temperature In Top M | Kelvin |
| **SLTYP** | Soil Texture Type By USDA | Category |

| Abb. | Variable Name (CMAQ) | Units |
| --- | --- | --- |
| NO2 | Nitrogen Di Oxide | ppmV |
| NO | Nitric Oxide | ppmV |
| O | Oxygen Atom | ppmV |
| O3 | Ozone | ppmV |
| NO3 | Nitrate | ppmV |
| O1D | Oxygen Atom | ppmV |
| OH | Hydroxide | ppmV |
| HO2 | Hydroperoxyl | ppmV |
| N2O5 | Nitrogen Pentoxide | ppmV |
| HNO3 | Nitric Acid | ppmV |
| HONO | Nitrous Acid | ppmV |
| H2O2 | Hydrogen Peroxide | ppmV |
| CO | Carbon Monoxide | ppmV |
| PAN | Peroxyacyl Nitrates | ppmV |

**References:**

1. Willmott, C. J. On the Validation of Models. *Phys. Geogr.* **2**, 184–194 (1981).

2. LeNail, A. NN-SVG: Publication-Ready Neural Network Architecture Schematics. *J. Open Source Softw.* **4**, 747 (2019).

3. Create Elegant Data Visualisations Using the Grammar of Graphics. https://ggplot2.tidyverse.org/.

4. Statistical Database | KOSIS KOrean Statistical Information Service. https://kosis.kr/eng/statisticsList/statisticsListIndex.do?menuId=M_01_01&vwcd=MT_ETITLE&parmTabId=M_01_01&statId=2008001&themaId=#SelectStatsBoxDiv.

5. Introduction — cartopy 0.18.0 documentation. https://scitools.org.uk/cartopy/docs/latest/.
